# Ophiostomatoid species associated with pine trees (*Pinus* spp.) infested by *Cryphaluspiceae* from eastern China, including five new species

**DOI:** 10.3897/mycokeys.83.70925

**Published:** 2021-10-13

**Authors:** Runlei Chang, Xiuyu Zhang, Hongli Si, Guoyan Zhao, Xiaowen Yuan, Tengteng Liu, Tanay Bose, Meixue Dai

**Affiliations:** 1 College of Life Sciences, Shandong Normal University, Jinan 250014, China Shandong Normal University Jinan China; 2 Kunyushan Forest Farm, Yantai 264112, China Kunyushan Forest Farm Yantai China; 3 Forestry and Agricultural Biotechnology Institute (FABI), Department of Biochemistry, Genetics & Microbiology, University of Pretoria, Pretoria 0002, South Africa University of Pretoria Pretoria South Africa

**Keywords:** *Ceratocystiopsis*, fungal symbionts, *Graphilbum*, nematode vector, *Ophiostoma*, *Sporothrix*

## Abstract

*Cryphaluspiceae* attacks various economically important conifers. Similar to other bark beetles, *Cr.piceae* plays a role as a vector for an assortment of fungi and nematodes. Previously, several ophiostomatoid fungi were isolated from *Cr.piceae* in Poland and Japan. In the present study, we explored the diversity of ophiostomatoid fungi associated with *Cr.piceae* infesting pines in the Shandong Province of China. We isolated ophiostomatoid fungi from both galleries and beetles collected from our study sites. These fungal isolates were identified using both molecular and morphological data. In this study, we recovered 175 isolates of ophiostomatoid fungi representing seven species. *Ophiostomaips* was the most frequently isolated species. Molecular and morphological data indicated that five ophiostomatoid fungal species recovered were previously undescribed. Thus, we proposed these five novel species as *Ceratocystiopsisyantaiensis*, *C.weihaiensis*, *Graphilbumtranslucens*, *Gr.niveum*, *and Sporothrixvillosa*. These new ophiostomatoid fungi add to the increasing number of fungi known from China, and this evidence suggests that numerous novel taxa are awaiting discovery in other forests of China.

## Introduction

Wingfield et al. (1993) coined the name “ophiostomatoid fungi” referring to a polyphyletic group of fungi that included several species from the orders *Microascales* and *Ophiostomatales*. These fungi are distinguished by spores generated in sticky droplets that aid in dispersion by arthropods (De Beer et al. 2013). The order *Microascales* includes three families, including *Ceratocystidaceae* (11 genera), *Gondwanamycetaceae* (2 genera), and *Graphiaceae* (1 genus) (De Beer et al. 2013). The *Ophiostomatales* was divided into two families: *Ophiostomataceae* (11 genera) and *Kathistaceae* (3 genera) (Hyde et al. 2020). Initially, De Beer and Wingfield (2013) identified 18 species complexes within the order *Ophiostomatales*. Later, the ‘*S.schenckii* – *O.stenoceras*’ species complex was elevated to genus level as *Sporothrix* (De Beer et al. 2016). Subsequently, this genus was divided into six species complexes (De Beer et al. 2016). Following this, Linnakoski et al. (2016a), Yin et al. (2016), and Jankowiak et al. (2017b) identified the *O.clavatum*, *O.piceae* and *G.grandifoliae* species complexes, respectively. Currently, the order *Ophiostomatales* thus encompasses at least 26 species complexes (De Beer and Wingfield 2013; De Beer et al. 2016; Linnakoski et al. 2016a; Yin et al. 2016; Jankowiak et al. 2017b).

Ophiostomatoid fungi often form a symbiotic association with bark and ambrosia beetles who assist in the dispersal of their inocula (Klepzig and Six 2004). For example, *Ceratocystiopsisranaculosus* colonizes the mycangium of *Dendroctonusfrontalis* whereas *Ophiostomaminus* is carried phoretically on the exoskeleton (Hofstetter et al. 2015). In addition, an ophiostomatoid fungus can symbiotically associate with multiple beetle species. Recently, six ophiostomatoid fungi were isolated from *Monochamusalternatus* in China (Zhao et al. 2014; Wang et al. 2018). Among them, *Ophiostomaips* was previously isolated from *Bursaphelenchusxylophilus* (Steiner & Buhrer) Nickle and *M.alternatus* Hope from North America and Korea, respectively (Wingfield 1987; Suh et al. 2013).

Beetle-associated ophiostomatoid fungi play pivotal roles in the ecosystem. As exemplified by *Endoconidiophorapolonica* and *Sporothrix* sp. 1., these fungi can provide beetles with nourishment, help them overcome plant defenses, and increase their vitality (Hammerbacher et al. 2013; Zhao et al. 2013; Wadke et al. 2016). *Endoconidiophorapolonica* uses plant defensive compounds such as stilbenes and flavonoids as a carbon source, whilst *Sporothrix* sp. 1. enhances the development and survival rate of arthropods such as *M.alternatus* (Zhao et al. 2013). This evidence confirms that ophiostomatoid fungi substantially influence the devastation caused by these arthropods in forestry contexts globally.

In Europe and Asia, *Cryphaluspiceae* infests various species of *Abies*, *Pinus*, *Picea*, and *Larix* (Jankowiak and Kolarik 2010). This bark beetle predominantly affects stressed trees (Michalski and Mazur 1999), but can also attack healthy ones (Justesen et al. 2020). Previously, several fungal species were isolated from *Cr.piceae* infesting *Abiesalba* and *A.veitchii*. This data included an assortment of ophiostomatoid fungi from the genera *Graphilbum*, *Grosmannia*, *Leptographium*, *Ophiostoma*, and *Sporothrix* from Poland (Jankowiak and Kolarik 2010; Jankowiak et al. 2017a) and Japan (Ohtaka et al. 2002a; Ohtaka et al. 2002b), and hypocrealean species from the genus *Geosmithia* from Poland (Jankowiak and Bilanski 2018).

In China, knowledge regarding the diversity of ophiostomatoid fungi associated with *Cr.piceae* is currently limited. Between 2019 and 2020, we thus conducted surveys of numerous *Pinus* stands in China’s Shandong province. During these surveys, we collected samples of wood and bark from afflicted trees that had beetle galleries. From these samples, 175 isolates of ophiostomatoid fungi were isolated. Analyses of molecular and morphological data revealed that our isolates belonged to seven different species of ophiostomatoid fungi. Among these, phylogenetic and morphological analyses confirmed that five of these taxa from China were previously undescribed. Here we described these species as *Ceratocystiopsisyantaiensis* sp. nov., *C.weihaiensis* sp. nov., *Graphilbumtranslucens* sp. nov., *Gr.niveum* sp. nov., and *Sporothrixvillosa* sp. nov.

## Materials and methods

### Collection of beetles and isolation of fungi

From September 2019 to August 2020, multiple surveys were conducted in several *Pinusthunbergii* stands located near Weihai (37°30'07"N, 121°07'24"E) and Yantai (37°15'38"N, 121°44'39"E), and *Pinusdensiflora* located near Qingdao (36°15'26"N, 121°38'07"E), Shandong Province of China. All these *Pinusthunbergii* and *Pinusdensiflora* stands were infested by *Cr.Piceae* along with *Bursaphelenchusxylophilus* and *Monochamusalternatus*. Samples of wood and bark with beetle galleries were collected from affected trees. In the laboratory, adult beetles from these galleries were individually collected in 2 ml sterile collection tubes inside a laminar flow cabinet. Both galleries and beetles were stored at 4 ℃ until the isolation of fungi.

Beetles were identified using both morphological and molecular data. In the case of the latter, cytochrome oxidase subunit I (COI) was used as the marker gene region. Sequences of bark beetle were identified using the “animal identification [COI]” database available through BOLDSYSTEMS (https://v3.boldsystems.org/). Sequence similarity searches confirmed the identity of all bark beetles as *Cr.piceae*. Hence, two representative sequences of the bark beetle were submitted to GenBank under the accession numbers MZ778788 and MZ778789.

In total, 32 adult beetles and 89 galleries were used for the isolation of ophiostomatoid fungi. Fungal isolation was done using the method suggested by Chang et al. (2019). Fungal mycelia and/or spore masses from *Cr.piceae* galleries were transferred onto 2% malt extract agar (MEA, Qingdao Hope Bio-technology, Qingdao, China) medium amended 0.05% streptomycin (Sangon Biotech, Qingdao, China). In cases of no mycelia and/or spore masses, galleries were incubated in moist chambers at 25 °C in darkness for 4–6 weeks. Post incubation, conidia with spore masses emerging from the conidiophores were transferred onto MEA amended with 0.05% streptomycin. To isolate ophiostomatoid fungi from the beetles, adult *Cr.piceae* was crushed on a sterile surface using a pair of forceps, thereafter, this crushed beetle was placed on the surface of MEA amended 0.05% streptomycin. To purify the fungal isolates, hyphal tips from fungal colonies were transferred onto fresh MEA plates.

All fungal isolates were submitted to the microbial culture collection of Shandong Normal University, Jinan, Shandong, China (SNM; for accession numbers see Table 1). Ex-holotypes cultures of ophiostomatoid fungi described in this study were deposited in the China General Microbiological Culture Collection Center (CGMCC; http://www.cgmcc.net/english/catalogue.html), Beijing, China. Holotype specimens (dry cultures) were deposited in the Herbarium Mycologicum, Academiae Sinicae (HMAS), Beijing, China.

**Table 1.** Isolates of ophiostomatoid fungi isolated from *Cryphaluspiceae* in this study.

Taxon	Species	Isolate	CGMCC	Tree host	Location	Sources	ITS	LSU	BT	EF	CAL
1	*Ceratocystiopsisyantaiensis* sp. nov.	SNM582		*Pinusthunbergii*	Yantai	Gallery	MW989410	MZ819923	MZ019522	MZ853079	–
		SNM650^T^	3.20247	*P.thunbergii*	Yantai	Gallyer	MW989411	MZ819924	MZ019523	MZ853080	–
2	*Ceratocystiopsisweihaiensis* sp. nov.	SNM634		*P.thunbergii*	Weihai	Gallery	MW989412	MZ819925	MZ019524	MZ853081	–
		SNM649^T^	3.20246	*P.thunbergii*	Weihai	Gallery	MW989413	MZ819926	MZ019525	MZ853082	–
3	*Graphilbumtranslucens* sp. nov.	SNM101		*P.thunbergii*	Weihai	Gallery	MW989414	–	MZ019526	MZ019544	MZ781969
		SNM104		*P.densiflora*	Qingdao	Gallery	MW989415	–	MZ019527	MZ019545	MZ781970
		SNM144^T^	3.20263	*P.thunbergii*	Weihai	Gallery	MW989416	–	MZ019528	MZ019546	MZ781971
4	*Graphilbumniveum* sp. nov.	SNM100		*P.densiflora*	Qingdao	Gallery	MW989417	–	MZ019529	MZ019547	MZ418998
		SNM145^T^	3.50268	*P.thunbergii*	Weihai	Beetle	MW989418	–	MZ019530	MZ019548	MZ418997
5	*Graphiumpseudormiticum*	SNM159		*P.thunbergii*	Weihai	Gallery	MW989419	–	–	MZ019549	–
6	*Ophiostomaips*	SNM20		*P.thunbergii*	Weihai	Gallery	MW989420	–	MZ019531	–	–
		SNM44		*P.thunbergii*	Weihai	Gallery	MW989421	–	MZ019532	–	–
		SNM110		*P.thunbergii*	Weihai	Gallery	MW989422	–	MZ019533	–	–
		SNM120		*P.thunbergii*	Weihai	Gallery	MW989423	–	MZ019534	–	–
		SNM121		*P.thunbergii*	Weihai	Gallery	MW989424	–	MZ019535	–	–
7	*Sporothrixvillosa* sp. nov.	SNM162		*P.thunbergii*	Weihai	Beetle	MW989425	–	MZ019536	MZ853075	MZ019540
		SNM182		*P.thunbergii*	Weihai	Beetle	MW989426	–	MZ019537	MZ853076	MZ019541
		SNM185		*P.thunbergii*	Weihai	Gallery	MW989427	–	MZ019538	MZ853077	MZ019542
		SNM188^T^	3.20264	*P.thunbergii*	Weihai	Beetle	MW989428	–	MZ019539	MZ853078	MZ019543

### DNA extraction, PCR amplification and sequencing

All fungal isolates obtained in this study were initially grouped based on colony morphology. For preliminary identification, at least two representative isolates from each group were identified using molecular techniques. For the novel species described in the present study, all isolates were sequenced to confirm their identity.

The PrepMan ultra sample preparation reagent (Applied Biosystems, Foster City, CA) was used for extracting the total genomic DNA from five-day-old cultures, following the manufacturer’s protocols. The complete ITS region, and partial large subunit (LSU) of the nuclear ribosomal RNA (rRNA) gene, and partial β-tubulin (BT), elongation factor 1-α (EF), and calmodulin (CAL) genes were amplified using primers ITS1F/ITS4 (White et al. 1990; Gardes and Bruns 1993), LR0R/LR5 (Vilgalys and Hester 1990), Bt2a (or T10)/Bt2b (Glass and Donaldson 1995), EF2F/EF2R (Jacobs et al. 2004; Marincowitz et al. 2015), and CL2F/CL2R (Duong et al. 2012), respectively.

Each 25 µl PCR reaction included 12.5 µl 2 × Taq Master Mix (buffer, dNTPs, and Taq; Vazyme Biotech Co., Ltd, China), 0.5 µl each of forward and reverse primers, 10.5 µl PCR grade water, and 1 µl of DNA template. PCR amplifications were conducted with an initial denaturation at 95 °C for 3 min, followed by 30 cycles of 95 °C for 60 sec; annealing temperature was 55 °C for 60 sec for all primers; 72 °C for 1 min; and final elongation at 72 °C for 10 min.

All PCR products were sequenced by Sangon Biotech, Qingdao, Shandong Province, China. The sequences were assembled using Geneious v. 7.1.4 (Biomatters, Auckland, New Zealand). The BLAST algorithm (Altschul et al. 1990) available through the NCBI GenBank was used for the preliminary identification of the taxa. All sequences were submitted to GenBank and the accession numbers are listed in Table 1.

### Phylogenetic analyses

For phylogenetic analyses, separate datasets were prepared for all four gene regions (ITS, BT, EF and CAL). Each of these datasets included sequences generated in this study, and those that were retrieved from the GenBank (including the ex-type sequences, Suppl. material 3: Table S1). We recovered a high number of isolates representing the same species from *O.ips* (141 isolates) and *S.gossypina* species complex (24 isolates). Therefore, datasets for these two species complexes included sequences from four representative isolates. The gene areas that are available in public databases substantially vary amongst genera and species complexes of ophiostomatoid fungi. As a result, we chose gene regions for our study based on previous research. These are as follows: ITS, BT, EF and CAT for *Graphilbum* (Jankowiak et al. 2020), ITS and BT for *O.ips* species complex (Wang et al. 2020), ITS, LSU and BT for *Ceratocystiopsis* (Nel et al. 2021), ITS, BT and CAL for *Sporothrix* (De Beer et al. 2016; Wang et al. 2018), and ITS and EF for *Graphium* (Chang et al. 2019). The datasets were aligned using MAFFT v. 7 (Katoh and Standley 2013). If needed, alignments were manually edited using MEGA v. 6.06 (Tamura et al. 2013). All aligned sequence datasets were deposited to TreeBase (Acc. No. 28127).

Programs used for maximum likelihood (ML) and Bayesian inference (BI) analyses were accessed through the CIPRES Science Gateway v. 3.3 (Miller et al. 2010). For all datasets, jModelTest v. 2.1.6 (Darriba et al. 2012) was used for selecting appropriate substitution models. Maximum likelihood analyses were done through RaxML v. 8.2.4 (Stamatakis 2014) using the GTR substitution model and 1000 bootstrap replicates. Bayesian inference analyses were done using MrBayes v. 3.2.6 (Ronquist et al. 2012). Four MCMC chains were run from a random starting tree for five million generations and trees were sampled every 100^th^ generation. One-fourth of the sampled trees were discarded as burn-in and the remaining trees were used for constructing majority rule consensus trees. MEGA-X was used for conducting maximum parsimony (MP) analyses with 1000 bootstrap replicates (Kumar et al. 2018) where gaps were treated as a fifth character.

### Growth and morphological studies

For each new fungal species, an ex-type along with another isolate identified through phylogenetic analyses were selected for growth study. Isolates were initially sub-cultured on 2% MEA and incubated for seven days at 25 °C in darkness. Thereafter, 5 mm agar plugs were placed at the center of 90 mm Petri dishes and three replicate plates per isolate were incubated at 5, 10, 15, 20, 25, 30 and 35 °C (± 0.5 °C) in darkness. The colony diameter of each isolate was measured at an interval of two days up to the tenth day.

Microscopic structures of the ophiostomatoid fungi were measured and photographed using a Zeiss Axio Imager Z2 (CarlZeiss, Germany). Fifty measurements for each taxonomically informative structure were made, such as conidiophore and conidia. The measurements are presented in the format (minimum–) mean minus standard deviation-mean plus standard deviation (–maximum).

## Results

### Collection of beetles and isolation of fungi

In the present study, 175 isolates of ophiostomatoid fungi were recovered. Among these, 148 were isolated from galleries, whereas 28 were from beetles. Based on the collection sites, 16, 63, and 96 isolates were recovered from Yantai, Qingdao, and Weihai, respectively.

### Phylogenetic analyses

Preliminary identification of the ophiostomatoid fungi recovered in this study showed that the isolates resided in the genera *Ceratocystiopsis* (4 isolates), *Graphilbum* (5 isolates), *Graphium* (1 isolate), *Ophiostoma* (141 isolates), and *Sporothrix* (24 isolates).

**Figure 1.**
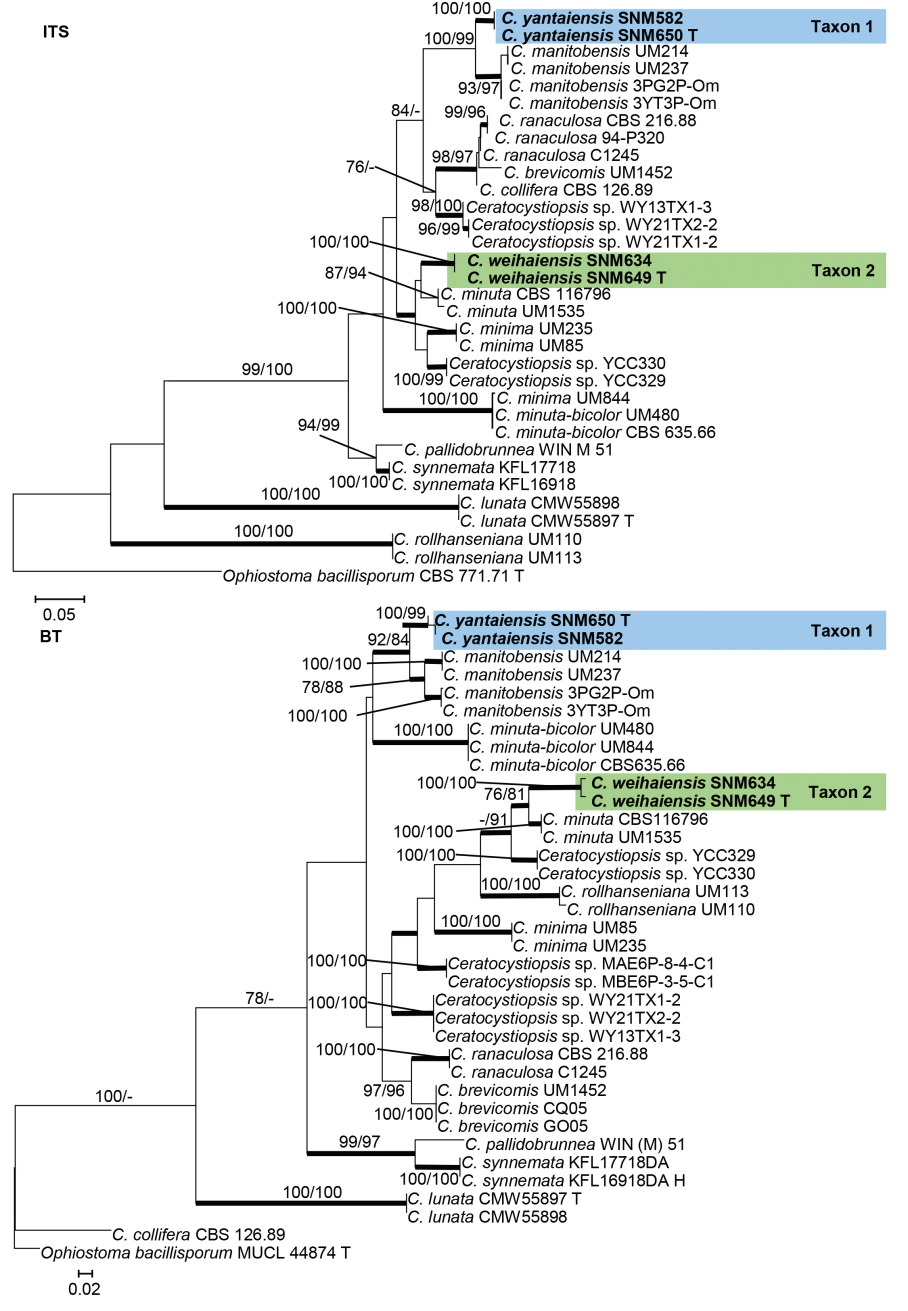
Maximum likelihood phylogeny of *Ceratocystiopsis* using complete ITS and partial BT gene regions. The isolates recovered in this study are highlighted in color and in bold font. ML and MP bootstrap support values ≥ 75 are indicated at the nodes as ML/MP. Bold branches indicate posterior probabilities values ≥ 0.9. T indicates ex-type cultures.

**Figure 2.**
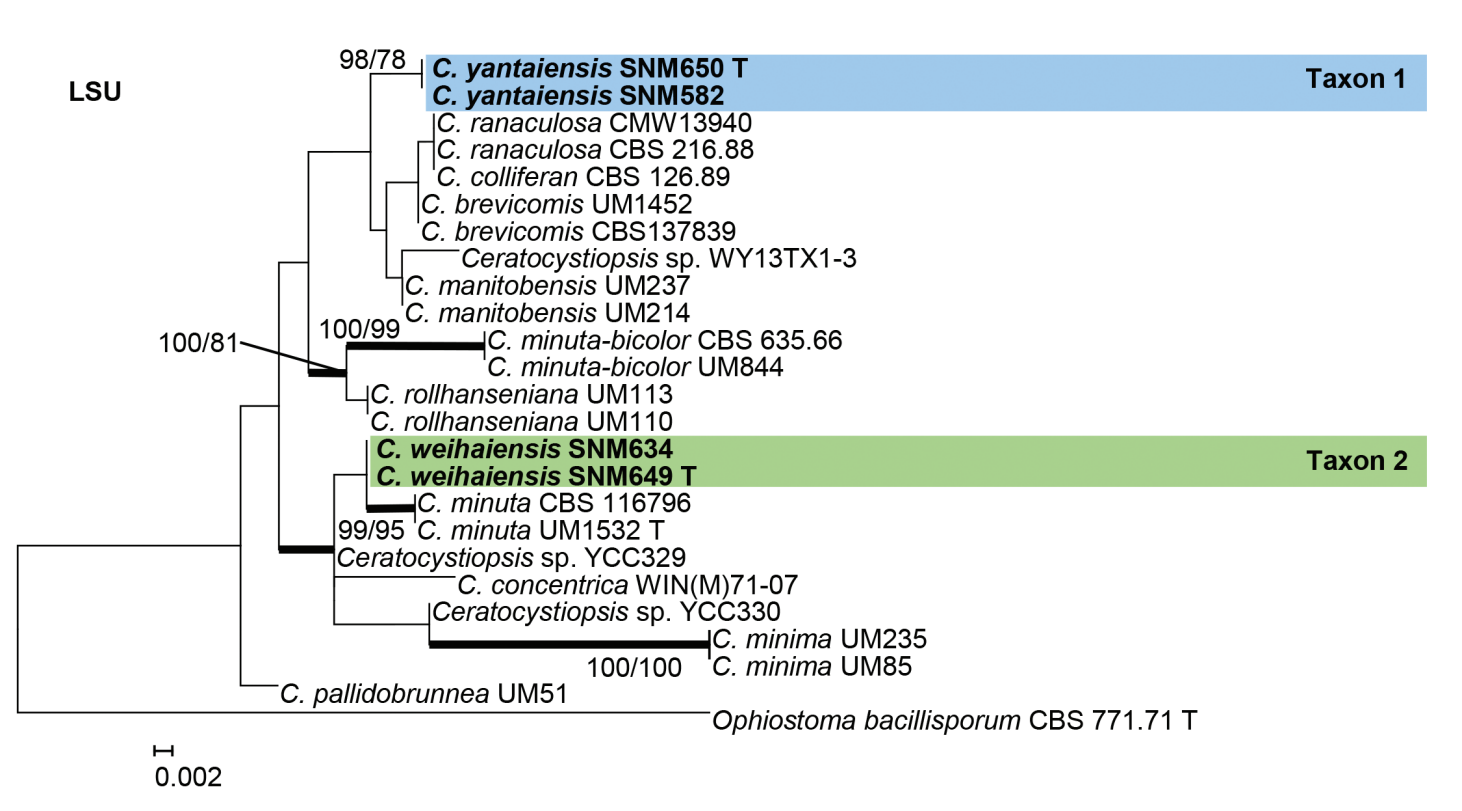
Maximum likelihood phylogeny of *Ceratocystiopsis* using partial LSU gene regions. The isolates recovered in this study are highlighted in color and in bold font. ML and MP bootstrap support values ≥ 75 are indicated at the nodes as ML/MP. Bold branches indicate posterior probabilities values ≥ 0.9. T indicates ex-type cultures.

Species residing in the genus *Ceratocystiopsis* were analyzed using ITS, LSU, and BT gene regions. In the phylogenies of *Ceratocystiopsis*, four isolates of *Ceratocystiopsis* clustered into two distinct monophyletic clades (Figs 1 and 2). Taxon 1 (two isolates) and Taxon 2 (two isolates) were found to be sisters to *C.manitobensis* and *C.minuta*, respectively (Figs 1 and 2).

**Figure 3.**
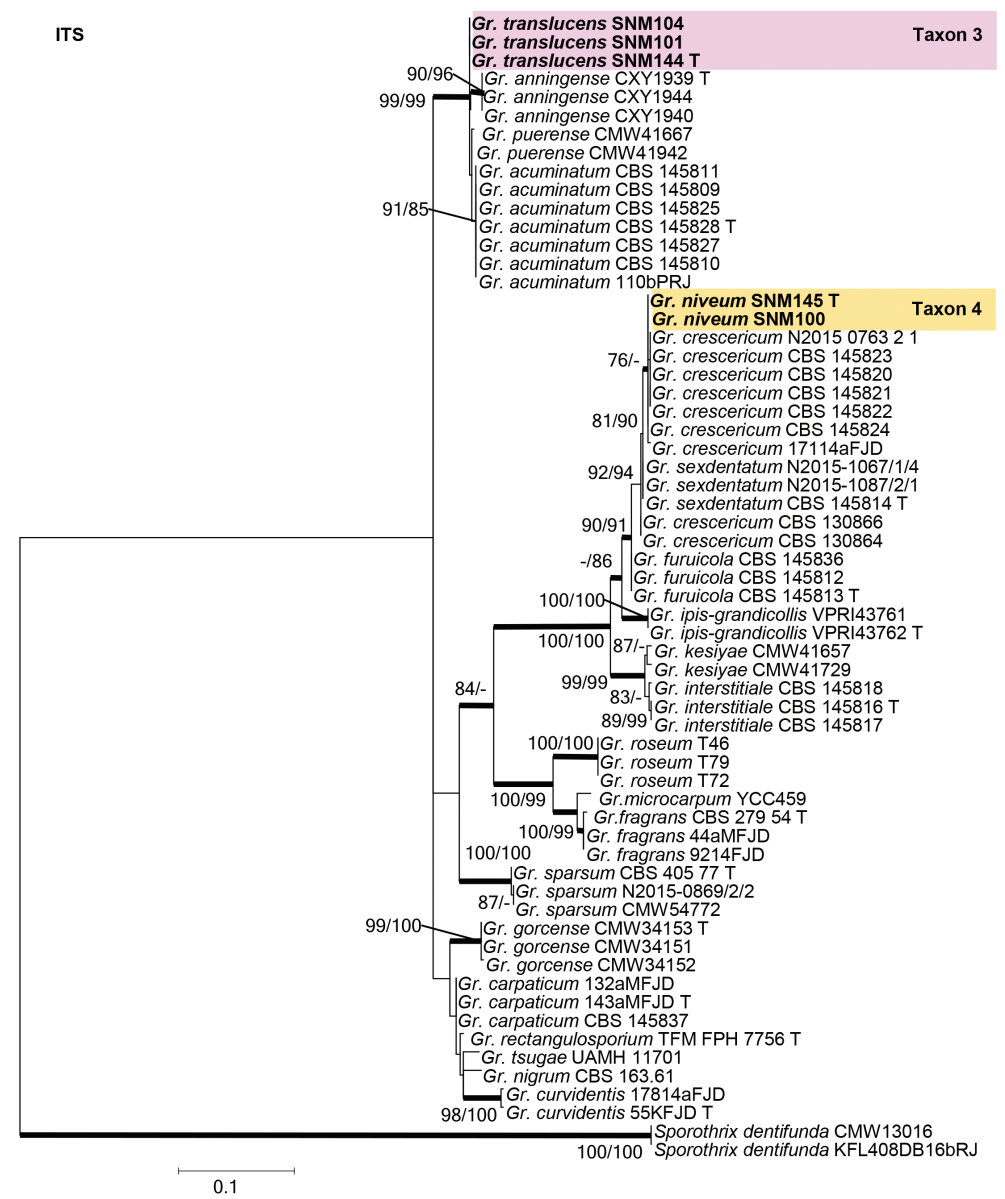
Maximum likelihood phylogeny of *Graphilbum* using complete ITS region. The isolates recovered in this study are highlighted in color and in bold font. ML and MP bootstrap support values ≥ 75 are indicated at the nodes as ML/MP. Bold branches indicate posterior probabilities values ≥ 0.9. T indicates ex-type cultures.

**Figure 4.**
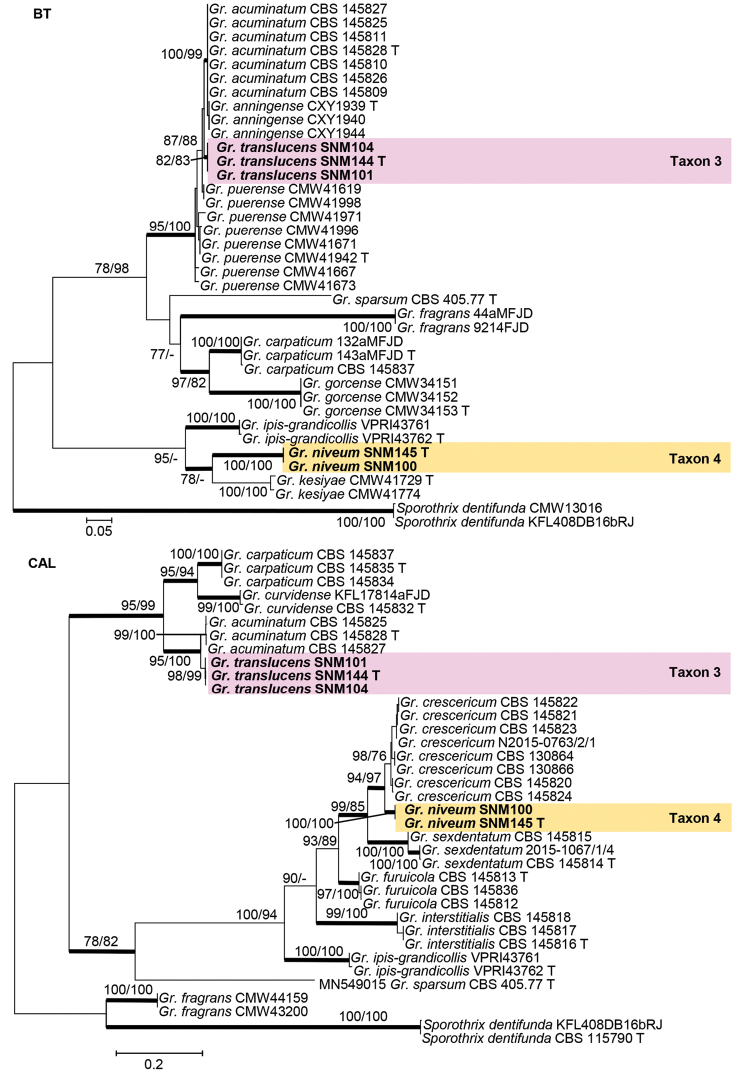
Maximum likelihood phylogeny of *Graphilbum* using partial BT and partial CAL gene regions. The isolates recovered in this study are highlighted in color and in bold font. ML and MP bootstrap support values ≥ 75 are indicated at the nodes as ML/MP. Bold branches indicate posterior probabilities values ≥ 0.9. T indicates ex-type cultures.

**Figure 5.**
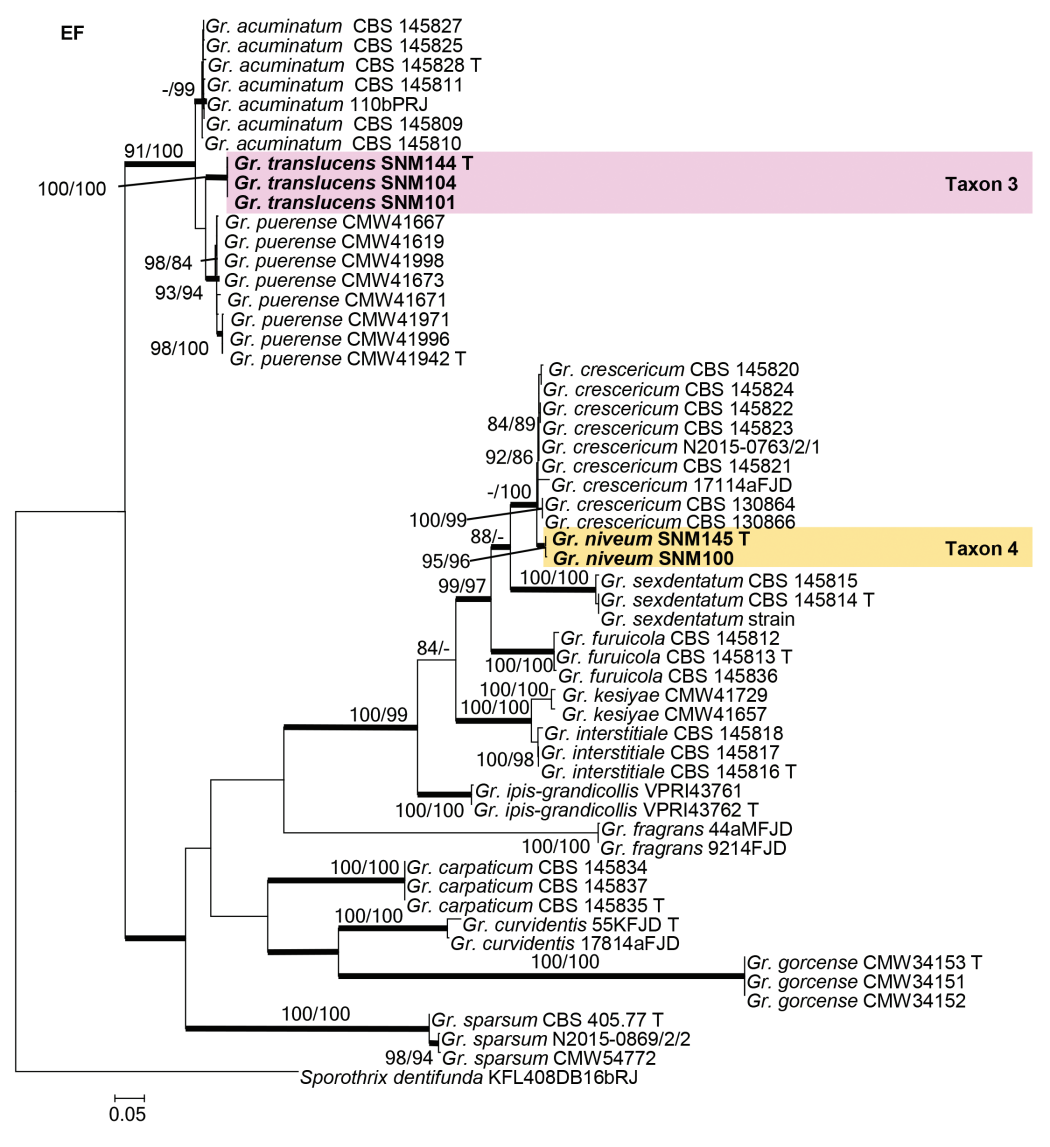
Maximum likelihood phylogeny of *Graphilbum* using partial EF gene region. The isolates recovered in this study are highlighted in color and in bold font. ML and MP bootstrap support values ≥ 75 are indicated at the nodes as ML/MP. Bold branches indicate posterior probabilities values ≥ 0.9. T indicates ex-type cultures.

Species residing in the genus *Graphilbum* were analyzed using ITS, BT, CAL, and EF gene regions. The taxon sampling differed substantially amongst the gene regions due to the lack of sequences. In the phylogenetic analyses, our five isolates of *Graphilbum* clustered into two distinct clades (Figs 3–5). The three isolates of Taxon 3 nested within clades that included *Gr.acuminatum*, *Gr.anningense*, and *Gr.puerense* (Figs 3–5). In the ITS, CAL, and EF trees, the two isolates of Taxon 4 were found to be closely related to *Gr.crescericum* (Figs 3–5). In contrast, Taxon 4 emerged as the sister species to *Gr.kesiyae* in the BT tree (Fig. 4). This is due to the lack of BT gene sequences for *Gr.crescericum*.

The identity of the isolate residing in *Graphium* was confirmed using ITS and EF gene regions. In the phylogenies, the single isolate of Taxon 5 emerged as a previously described species, *G.pseudormiticum* (Suppl. material 1).

Species resided in the *O.ips* species complex were analyzed using ITS and BT gene regions. In the ITS and BT trees, our isolates of Taxon 6 (141 isolates) formed monophyletic clades with *O.ips* (Suppl. material 2).

**Figure 6.**
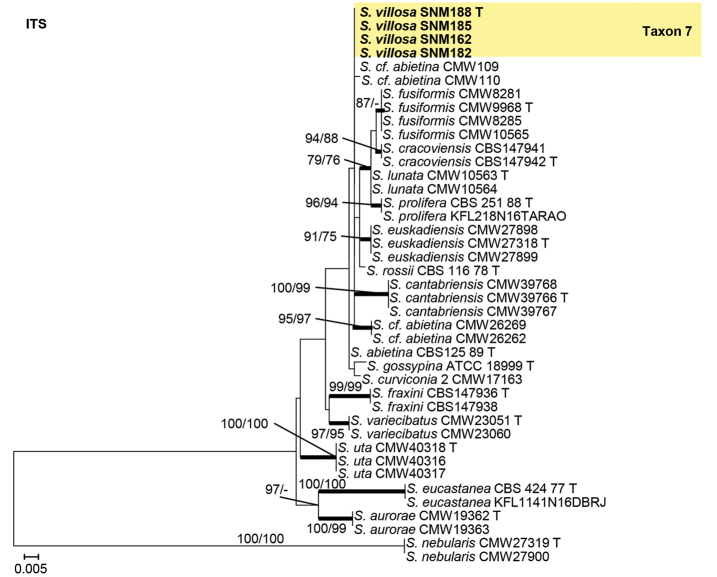
Maximum likelihood phylogeny of *Sporothrixgossypina* species complex using complete ITS region. The isolates recovered in this study are highlighted in color and in bold font. ML and MP bootstrap support values ≥ 75 are indicated at the nodes as ML/MP. Bold branches indicate posterior probabilities values ≥ 0.9. T indicates ex-type cultures.

**Figure 7.**
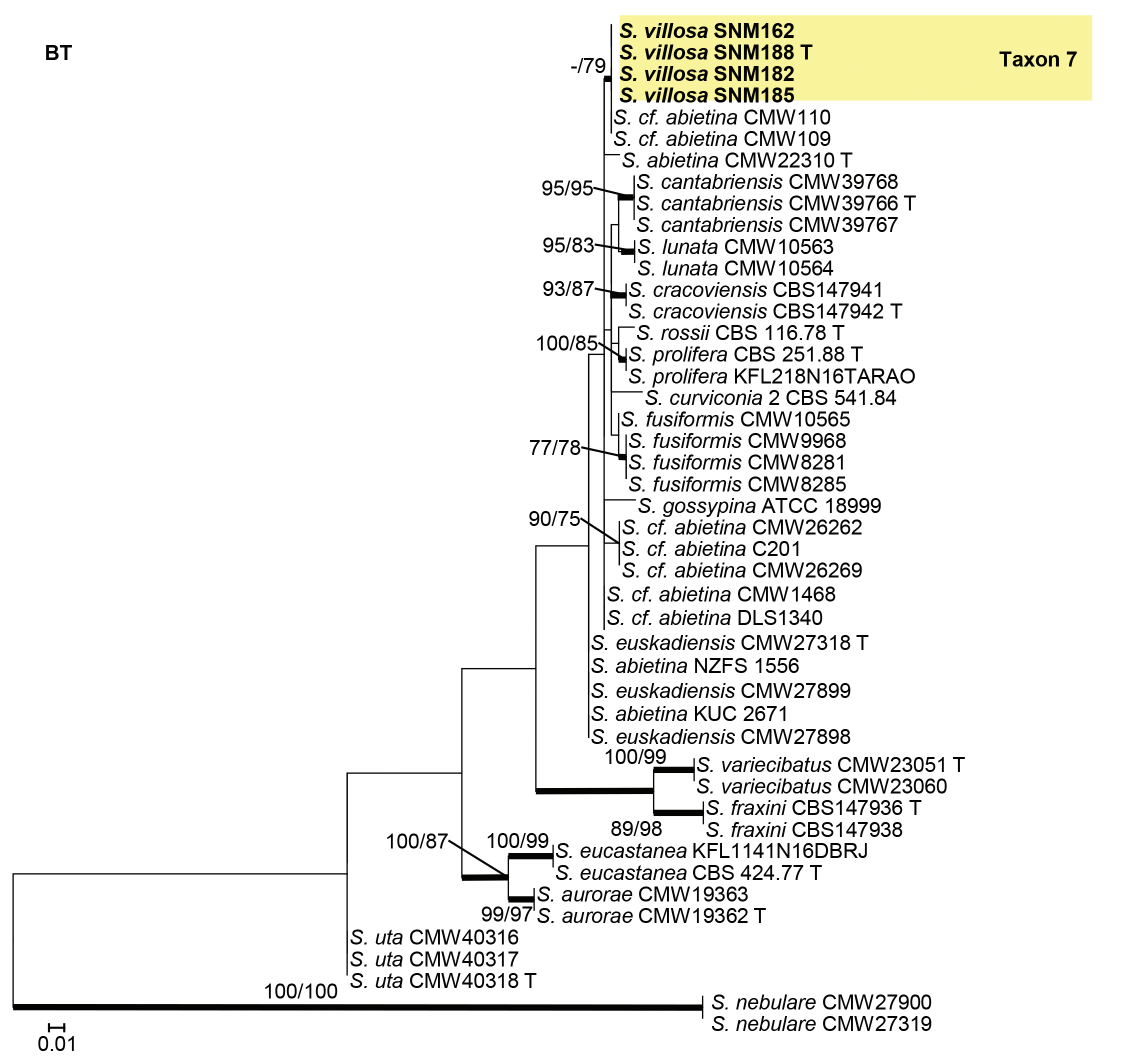
Maximum likelihood phylogeny of *Sporothrixgossypina* species complex using partial BT gene region. The isolates recovered in this study are highlighted in color and in bold font. ML and MP bootstrap support values ≥ 75 are indicated at the nodes as ML/MP. Bold branches indicate posterior probabilities values ≥ 0.9. T indicates ex-type cultures.

**Figure 8.**
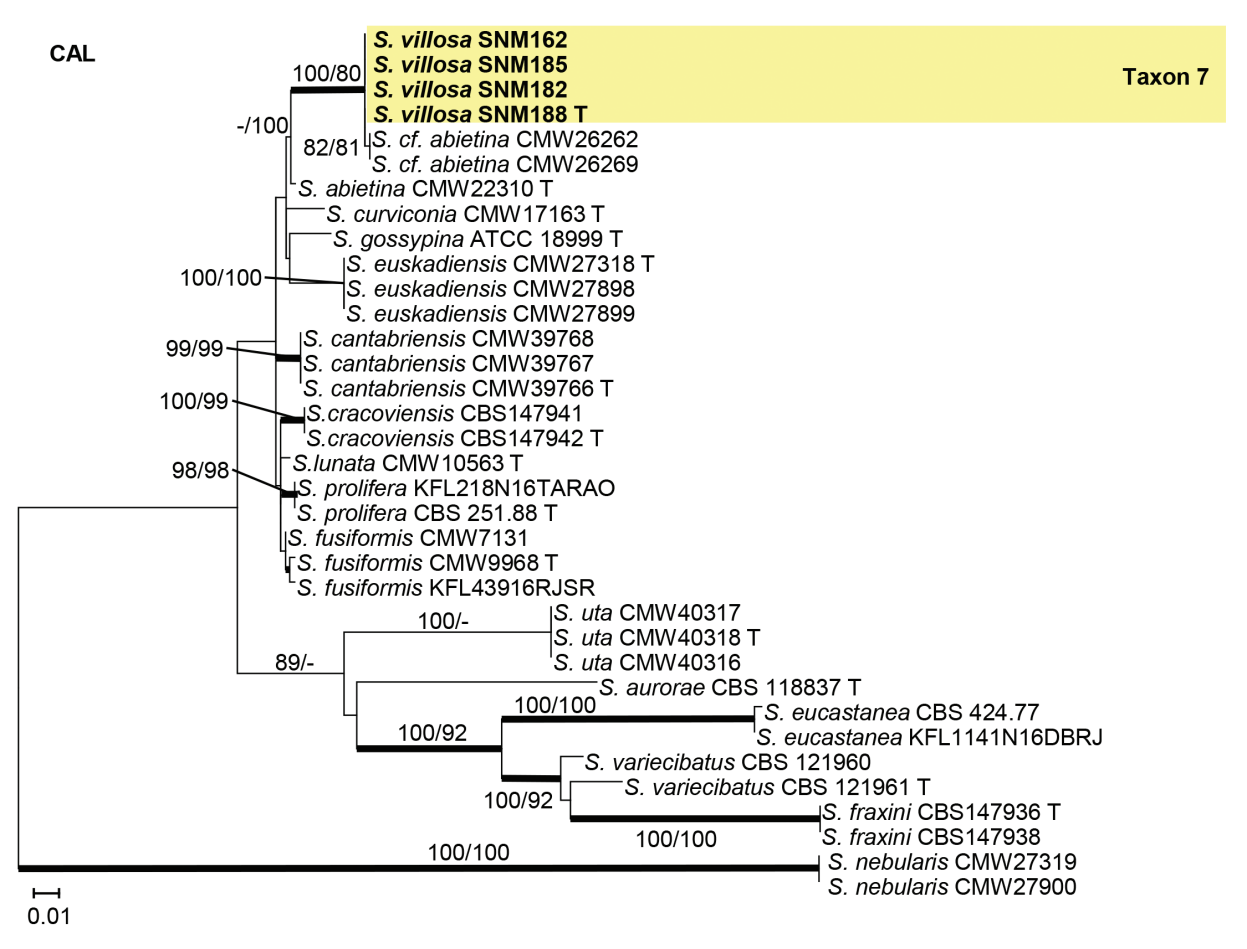
Maximum likelihood phylogeny of *Sporothrixgossypina* species complex using partial CAL gene region. The isolates recovered in this study are highlighted in color and in bold font. ML and MP bootstrap support values ≥ 75 are indicated at the nodes as ML/MP. Bold branches indicate posterior probabilities values ≥ 0.9. T indicates ex-type cultures.

Isolates from the *S.gossypina* species complex were analyzed using ITS, BT, and CAL gene regions. In the phylogenetic analyses, our isolates of Taxon 7 were found to be closely related to two fungal isolates from China that were previously identified as *S.cf.abietina* (Figs 6–8).

### Taxonomy

#### 1. Ceratocystiopsis yantaiensis

Taxon classificationFungiOphiostomatalesOphiostomataceae

R.L. Chang & X.Y. Zhang sp. nov.

18C2E7BC-1929-5A08-95DA-3A1647FF5A1F

839252

Fig. 9

##### Holotype.

China. Shandong province: Kunyushan National Forest Park, Yantai city, from the gallery of *Cryphaluspiceae* on *Pinusthunbergii*, 2 Sep. 2020, R. L. Chang (HMAS249924-holotype; SNM650 = CGMCC3.20247 – ex-holotype culture).

##### Additional cultures checked.

China. Shandong province: Kunyushan National Forest Park, Yantai city, from the gallery of *Cryphaluspiceae* on *Pinusthunbergii*, 2 Sep. 2020, R. L. Chang (SNM582).

##### Etymology.

The name refers to Yantai City, where this fungus was isolated.

##### Diagnosis.

*Ceratocystiopsisyantaiensis* differs from closely related species by the production of smaller conidia.

##### Description.

**Figure 9.**
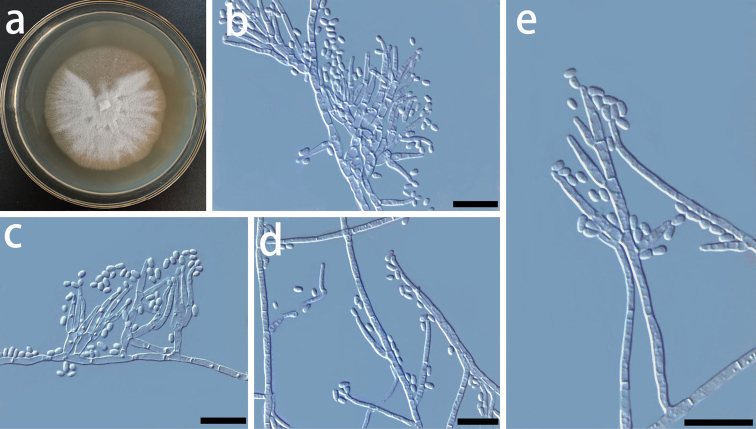
Morphological characters of asexual structures of *Ceratocystiopsisyantaiensis* sp. nov. **a** fourteen-day-old culture on MEA**b, c** type 1 conidiophores and conidia **d-e** type 2 conidiophores and conidia. – Scale bars: 10 μm.

Sexual morph is unknown. Asexual state hyalorhinocladiella-like: the conidiophores directly arising singly from the vegetative hyphae, measuring (2.4–) 4.7–26.7 (–46.4) μm × (0.8–) 1.0–1.5 (–1.8) μm (Fig. 9d, e); or a short basal cell which continues to develop short lateral and terminal extensions from conidiogenous sites at their apices or discrete basal cells that produce 1–5 branches, which then branch irregularly and form conidiogenous cells at their apices, measuring (12.2–) 6.2–10.2 (–50.7) μm long (Fig. 9b, c); conidiogenous cells measuring (4.7–) 6.2–10.2 (–12.4) × (0.7–) 0.9–1.3(–1.5) μm (Fig. 9b, c); conidia hyaline, smooth, unicellular, short oblong, with rounded ends, measuring (1.1–) 1.4–2.2 (–2.7) × (0.8–) 0.9–1.2 (–1.5) μm (Fig. 9b-e).

##### Culture characteristics.

The Colonies are light brown in color on MEA (Fig. 9a). Mycelia are white, superficially growing on the agar. The optimal temperature for growth was 30–35 °C, reaching 43.0 mm diam in 10 days. No growth was observed at 5 °C.

##### Distribution.

Currently known from Yantai City in Shandong Province, China.

##### Note.

*Ceratocystiopsisyantaiensis* is phylogenetically close to *C.manitobensis*, but formed a distinct clade on both ITS, LSU, and BT trees (Figs 1 and 2). Two types of hyalorhinocladiella-like asexual state were also observed in *C.manitobensis* (Hausner et al. 2003). Conidia of *C.yantaiensis* and *C.manitobensis* are similar in morphology, but there is a difference in size (1.1–2.7 × 0.8–1.5 vs. 3.0–5.5 × 1.0–2.0 µm, Fig. 9b-e).

#### 2. Ceratocystiopsis weihaiensis

Taxon classificationFungiOphiostomatalesOphiostomataceae

R.L. Chang & X.Y. Zhang sp. nov.

1052D7A3-02F8-5BA7-B252-394CA55C5C4C

839253

Fig. 10

##### Holotype.

China. Shandong province: Zhujiajuan village, Huancui District, Weihai City, from the gallery of *Cryphaluspiceae* on *Pinusthunbergii*, 2 Sep. 2019, R. L. Chang (HMAS 249923-holotype; SNM649 = CGMCC3.20246 – ex-holotype culture).

##### Additional cultures checked.

China. Shandong province: Zhujiajuan village, Huancui District, Weihai City, from the gallery of *Cryphaluspiceae* on *Pinusthunbergii*, 2 Sep. 2019, R. L. Chang (SNM634).

##### Etymology.

The name refers to Weihai City, where this fungus was isolated.

##### Diagnosis.

Compared to other closely related species, the conidia of *C.weihaiensis* are smaller.

##### Description.

**Figure 10.**
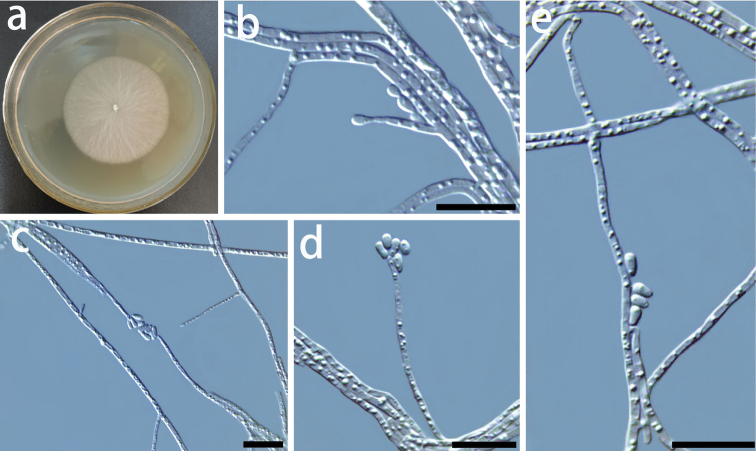
Morphological characters of asexual structures of *Ceratocystiopsisweihaiensis* sp. nov **a** fourteen-day-old culture on MEA**b-e** conidiophores and conidia. – Scale bars: 10 μm.

Sexual morph is unknown. Asexual state hyalorhinocladiella-like: the conidiophores directly arise singly from the vegetative hyphae, measuring (2.6–) 10.9–29.2 (–44.6) μm × (0.7–) 0.9–1.3 (–1.6) μm (Fig. 10b-e); conidia hyaline, smooth, unicellular short oblong, with rounded ends or clavate, ellipsoidal to ovoid measuring (1.5–) 2.0–2.6 (–2.9) × (0.7–) 0.9–1.2 (–1.5) μm (Fig. 10b-e).

##### Culture characteristics.

The colonies are light brown in color on MEA (Fig. 10a). Mycelia white, submerged in the agar. The optimal temperature for growth is 30 °C, reaching 46.0 mm diam in 10 days. Growth is slower at 35 °C, 27 mm diam in 10 days.

##### Distribution.

Currently known from Weihai City in Shandong Province, China.

##### Note.

*Ceratocystiopsisweihaiensis* is phylogenetically close to *C.minuta*, but formed a distinct monophyletic clade on both ITS and BT trees (Figs 1 and 2). In the phylogenetic study of *C.minuta* by Plattner et al. (2009) using ITS, LSU, and BT gene regions, the authors suggested that this taxon is possibly an assemblage of multiple species. Therefore, they designated the strain RJ705 from Poland as the neotype. Later, strain RJ705 = UAMH 11218 = WIN(M) 1532 was considered as the lectotype for *C.minuta* (Reid and Hausner 2010).

*Ceratocystiopsisminuta* and most other *Ceratocystiopsis* species have a hyalorhinocladiella-like asexual state (Plattner et al. 2009; De Beer and Wingfield 2013). The conidia of *C.weihaiensis* and *C.minuta* are similar in gross morphology. The *C.weihaiensis* differs from *C.minuta* in having short conidia size (1.5–2.9 × 0.7–1.5 vs. 2–4 × 1–2 μm, Fig. 10b-e) (Reid and Hausner 2010).

#### 3. Graphilbum translucens

Taxon classificationFungiOphiostomatalesOphiostomataceae

R.L. Chang & X.Y. Zhang sp. nov.

0AD17C86-5151-538F-AFB5-F1BE7F7C8BE7

839254

Fig. 11

##### Holotype.

China. Shandong province: Zhujiajuan village, Huancui District, Weihai City, from the gallery of *Cryphaluspiceae* on *Pinusthunbergii*, 10 Oct. 2019, R. L. Chang (HMAS 249925-holotype; SNM144 = CGMCC 3.20263 – ex-holotype culture).

##### Additional cultures checked.

China. Shandong province: Laojiangou village, Laoshan District, Qingdao City, from the gallery of *Cryphaluspiceae* on *Pinusdensiflora*, 2, Aug. 2020, R. L. Chang (SNM104).

##### Etymology.

The name refers to the translucent appearance of the colony on MEA.

##### Diagnosis.

*Graphilbumtranslucens* can be distinguished from other closely related species, *Gr.puerense* and *Gr.acuminatum*, by the shorter hyalorhinocladiella-like conidiophores, smaller conidia and no pesotum-like asexual state.

##### Description.

**Figure 11.**
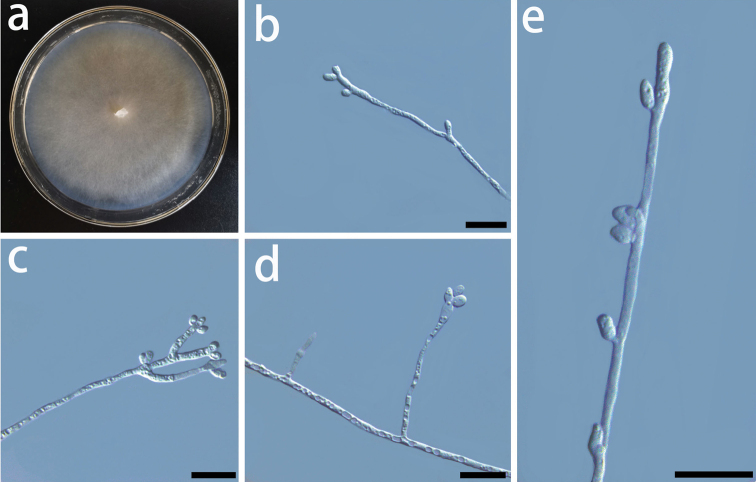
Morphological characters of asexual structures of *Graphilbumtranslucens* sp. nov. **a** fourteen-day-old culture on MEA**b-e** conidiophores and conidia. – Scale bars: 10 μm.

Sexual morph is unknown. Asexual state hyalorhinocladiella-like: the conidiophores directly arising from the vegetative hyphae, measuring (3.6–) 8.6–42.2 (–72.3) μm × (0.9–) 1.1–1.7 (–2.0) μm (Fig. 11b-e); conidia hyaline, smooth, unicellular short oblong, with rounded ends or ellipsoidal to ovoid, measuring (2.1–) 2.4–3.5 (–4.1) × (0.8–) 1.3–2.0 (–2.7) μm (Fig. 11b-e).

##### Culture characteristics.

The colonies are light brown in color on MEA (Fig. 11a). Mycelia are partially submerged in the agar. The optimal temperature for growth is 30 °C, reaching 74.0 mm diam in 5 days. Growth slower at 35°C, 24 mm diam in 5 days. No growth was observed at 5 °C.

##### Distribution.

Currently known from Qingdao City and Weihai City in Shandong Province, China.

##### Note.

Based on morphology coupled with single-gene (ITS, EF, BT, and CAL) phylogenies, *Graphilbumtranslucens* is phylogenetically close to *Gr.puerense* and *Gr.acuminatum*. In the ITS tree, *Gr.translucens* grouped with *Gr.puerense* (Fig. 3) and *Gr.acuminatum* whereas it formed distinct clades in the BT and EF trees (Figs 4 and 5). The hyalorhinocladiella-like asexual state was observed in *Gr.translucens* and *Gr.puerense*, but it is absent in *Gr.acuminatum* (Chang et al. 2017; Jankowiak et al. 2020). The conidiophores of *Gr.translucens* are shorter than the *Gr.puerense* (Chang et al. 2017). Conidia of *Gr.translucens* and *Gr.puerense* form hyalorhinocladiella-like asexual states that are similar in shape, yet the conidia size of *Gr.translucens* is smaller than *Gr.puerense* (2.1–4.1 × 0.8–2.7 vs. 3.5–12 × 1–3 μm, Fig. 11b-e) (Chang et al. 2017). Unlike *Gr.puerense* and *Gr.acuminatum*, a pesotum-like asexual state was not observed among the isolates of *Gr.translucens* recovered in this study.

#### 4. Graphilbum niveum

Taxon classificationFungiOphiostomatalesOphiostomataceae

R.L. Chang & X.Y. Zhang sp. nov.

E5D2A04A-AF94-5EF9-AF13-396F00BE7527

840197

Fig. 12

##### Holotype.

China. Shandong province: Zhujiajuan village, Huancui District, Weihai City, from *Cryphaluspiceae* on *Pinusthunbergii*, 10 Oct. 2019, R. L. Chang (HMAS 350268-holotype; SNM145 = CGMCC3.20423– ex-holotype culture).

##### Additional cultures checked.

China. Shandong province: Laojiangou village, Laoshan District, Qingdao City, from the gallery of *Cryphaluspiceae* on *Pinusdensiflora*, 2, Aug. 2020, R. L. Chang (SNM100).

##### Etymology.

The name refers to the white mycelia that appear on the MEA after 14 days.

##### Diagnosis.

*Graphilbumniveum* differs from the closely related species *Gr.crescericum* by its shorter conidiophore and conidia.

##### Description.

**Figure 12.**
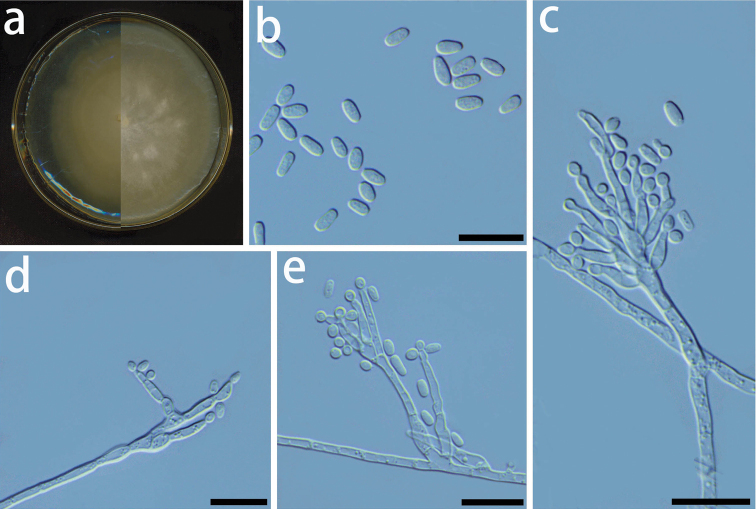
Morphological characters of asexual structures of *Graphilbumniveum* sp. nov. **a** left: seven-day-old culture on MEA; right: twenty-day-old culture on MEA**b***Conidia***c-e** conidiophores and conidia. – Scale bars: 10 μm.

Sexual morph is unknown. Asexual state hyalorhinocladiella-like: the conidiophores directly arising from the vegetative hyphae, or produce 1–3 branches, which then branch irregularly and form conidiogenous cells at their apices, measuring (14.0–) 21.7–36.7 (–56.0) μm (Fig. 12c-e); conidiogenous cell hyaline, discrete, measuring (6.2–)8.4–13.8 (–18.7) μm × (0.7–) 0.9–1.3 (–1.8) μm (Fig. 12c -e); conidia hyaline, smooth, unicellular oblong to ovoid, with rounded ends, measuring (2.2–) 2.6–3.4 (–4.1) × (0.8–) 1.0–1.6 (–1.8) μm (Fig. 12b-e).

##### Culture characteristics.

Colonies at first translucent to light brown in color on MEA (7 days). Thereafter, turning white in colour after 14 days (Fig. 12a). Mycelia are partially submerged in the agar. The optimal temperature for growth is 25 °C, reaching 61.0 mm diam in 8 days. The growth is relatively slower at 5 and 35 °C, reaching 2.7 mm and 9.1 mm diam in 8 days, respectively.

##### Distribution.

Currently known from Qingdao and Weihai City in Shandong Province, China.

##### Note.

Phylogenetic analyses based on each ITS, EF, and CAL tree shows that *Gr.niveum* is phylogenetically close to *Gr.crescericum* (Figs 3–5). In the ITS tree (Fig. 3), *Gr.niveum* clustered with *Gr.crescericum* whereas they a distinct clade in the EF and CAL trees (Figs 4 and 5). In both these species, the asexual structure is hyalorhinocladiella-like. Nonetheless, the conidiophore of *Gr.niveum* is shorter than *Gr.crescericum* (14.0–56.0 vs. 16.3–69.9 μm) (Romón et al. 2014b). Additionally, the conidia of *Gr.niveum* and *Gr.crescericum* are similar in shape, but differ in sizes. The conidia of *Gr.niveum* (2.2–4.1 × 0.8–1.8 µm) are substantially smaller than those of *Gr.crescericum* (4.4–6.2 × 1.7–3.3 μm). Furthermore, the colony color of *Gr.niveum* is light brown at first, whereas that of *Gr.crescericum* is white (Romón et al. 2014b).

*Graphilbumniveum* emerged as a sister to *Gr.kesiyae* in the BT tree. This is because sequences for the BT gene region were unavailable for *Gr.crescericum*. *Graphilbumkesiyae* has both pesotum-like and hyalorhinocladiella-like asexual states, whereas *Gr.niveum* exclusively has the latter one. Furthermore, *Gr.niveum*’s conidiogenous cells and conidia are smaller than those of *Gr.kesiyae* (Chang et al. 2017).

#### 7. Sporothrix villosa

Taxon classificationFungiOphiostomatalesOphiostomataceae

R.L. Chang & X.Y. Zhang sp. nov.

338DB4B6-3C8D-5916-9EB3-3D44995BFD7D

839255

Fig. 13

##### Holotype.

China. Shandong province: Zhujiajuan village, Huancui District, Weihai City, from *Cryphaluspiceae* on *Pinusthunbergii*, 10 Oct. 2019, R. L. Chang (HMAS 249926-holotype; SNM188 = CGMCC 3.20264– ex-holotype culture).

##### Additional cultures checked.

China. Shandong province: Zhujiajuan village, Huancui District, Weihai City, from *Cryphaluspiceae* on *Pinusthunbergii*, 10 Oct. 2019, R. L. Chang (SNM162); China. Shandong province: Zhujiajuan village, Huancui District, Weihai City, from *Cryphaluspiceae* on *Pinusthunbergii*, 10 Oct. 2019, R. L. Chang (SNM182).

##### Etymology.

The name refers to the velvety colony morphology of this fungus on MEA.

##### Diagnosis.

*Sporothrixvillosa* differ from *S.abietina* by the production of smaller conidia and slow growth rate on MEA at 35 °C.

##### Description.

**Figure 13.**
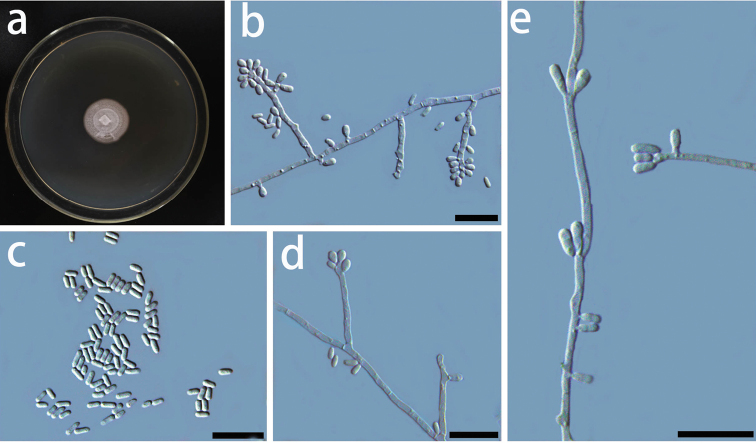
Morphological characters of asexual structures of *Sporothrixvillosa* sp. nov. **a** fourteen-day-old culture on MEA**b-e** conidiophores and conidia. – Scale bars: 10 μm.

Sexual morph is unknown. Asexual state sporothrix-like: the conidiophores directly arising from the vegetative hyphae, measuring (3.2–) 6.8–23.8 (–53.6) μm × (0.5–) 0.8–1.3 (–1.5) μm (Fig. 13b, d and e); conidia hyaline, smooth, unicellular oblong to ovoid, with rounded ends, measuring (1.2–) 1.8–2.6 (–4.1) × (0.7–) 0.8–1.1 (–1.4) μm (Fig. 13c).

##### Culture characteristics.

The colonies are white in color on MEA. Mycelia were submerged in the agar. The optimal temperature for growth is 25 °C, reaching 21.1 mm diam in 10 d. Growth is extremely slow at 35°C 3 mm diam in 10 days. No growth was observed at 5 °C.

##### Distribution.

Currently known from Weihai City in Shandong Province, China.

##### Note.

*Sporothrixvillosa* is closely related to two fungal isolates recovered from China in CAL tree, and another two isolates recovered from the USA in ITS and BT trees, which were previously identified as *S.cf.abietina*. This taxon is phylogenetically distinct from all other species in the *S.gossypina* species complex (Figs 6–8). Six et al. (2011) classified all the isolates from China, Canada, the USA, New Zealand, Korea, and South Africa that were close to the ex-type cultures on the BT tree as *S.abietina*. However, these selected isolates did not form a monophyletic clade. Later, in the phylogenies using BT and CAL gene-regions, these isolates of *S.abietina* did not cluster with the ex-type isolates of *S.abietina*. Therefore, these isolates were provisionally identified as *S.cf.abietina* (Romón et al. 2014a; Romón et al. 2014b). Our phylogenetic analyses indicated that isolates classified as *S.abietina* (Six et al. 2011) plausibly included several phylogenetic distinct species. In this study, *Sporothrixvillosa* recovered produced a sporothrix-like asexual morph similar to other species in the complex. Furthermore, the conidia of *S.villosa* (Fig. 13c) are smaller than those of *S.abietina* (1.2–4.1 × 0.7–1.4 vs. 4–7.5 × 1–2 μm) (Marmolejo and Butin 1990). Unlike *S.abietina*, *S.villosa* can grow slowly at 35 °C.

## Discussion

In the present study, we collected *Cryphaluspiceae* and their galleries from various pine forests located near Qingdao, Weihai, and Yantai cities in the Shandong province of China. From these beetles and galleries, we recovered 175 isolates of ophiostomatoid fungi representing seven well-defined genera. These genera were *Ceratocystiopsis*, *Graphilbum*, *Graphium*, *Ophiostoma*, and *Sporothrix*. Based on molecular and morphological data, the data indicated that five of the ophiostomatoid fungal species recovered in this study were previously undescribed. Hence, we newly described these ophiostomatoid species as *C.yantaiensis*, *C.weihaiensis*, *Gr.translucens*, *Gr.niveum*, and *S.villosa*.

*Ophiostomaips* was one of the most frequently isolated ophiostomatoid fungi in China and this study (Lu et al. 2009; Chang et al. 2017; Wang et al. 2018; Chang et al. 2019). Across China, this fungus was also found associated with various species of mites and bark beetles (Chang et al. 2017). As reported for *Sporothrix* sp.1, in the symbiotic relationship between *M.alternatus*-*B.xylophilus*-ophiostomatoid fungi, *O.ips* substantially influences the survival and reproduction of the other two partners (Niu et al. 2012; Zhao et al. 2013). Earlier, *O.ips* was also isolated from *M.alternatus*, but its specific function in this symbiotic relationship is still unknown (Zhao et al. 2018). Therefore, it is not unreasonable to hypothesize that this symbiotic fungus also influences the life history and population of its vector and associated nematode.

*Cryphaluspiceae* vectors diverse groups of fungi and nematodes. At least sixty fungal species have been found associated with this beetle. Globally, the diversity of fungi that are associated with *Cr.piceae* varies greatly (Ohtaka et al. 2002a; Ohtaka et al. 2002b; Jankowiak and Kolarik 2010; Jankowiak et al. 2017a; Jankowiak and Bilanski 2018). In Europe, several *Geosmithia* species were found associated with *Cr.piceae* (Jankowiak and Kolarik 2010; Kolařík and Jankowiak 2013; Jankowiak and Bilanski 2018). However, we did not recover any *Geosmithia* in this study. In Poland and Japan, the most frequently isolated ophiostomatoid fungi derived from *Cr.piceae* was *O.piceae*, *Leptographiumeurophioides* and *O.subalpinum*, respectively (Ohtaka et al. 2002b; Yamaoka et al. 2004; Jankowiak and Kolarik 2010). However, in our study, the dominant fungal species was *O.ips*. A similar trend was also reported from other ophiostomatoid fungi-bark beetle relationships, such as those with *Ips typographus* and *Dendroctonusvalens* (Taerum et al. 2013; Chang et al. 2019). This data suggests that the relationship between bark beetles and their fungal associates is casual.

This shift in the diversity of ophiostomatoid fungi that are associated with bark beetles is possibly influenced by both climatic factors and host tree species. Previously, Linnakoski et al. (2016b) indicated that temperature can significantly influence the diversity of fungi that are associated with bark beetles. This is not an unreasonable hypothesis because the climatic conditions in China, Japan, and Poland are considerably different, which may influence the fungal diversity associated with various species of bark beetles from these regions. In China, we isolated these ophiostomatoid fungi from *Cr.piceae* infecting pine trees, whereas in Japan and Poland, hosts included various species of *Abies* (Ohtaka et al. 2002a; Ohtaka et al. 2002b; Yamaoka et al. 2004; Jankowiak and Kolarik 2010). Besides climate, this difference in the host tree species could have also influenced the diversity of symbiotic fungi associated with *Cr.piceae*.

Ophiostomatoid fungi are an enigmatic taxonomic group (De Beer et al. 2013). As reported previously and in the present study, the morphological differences between the species are often slim (De Beer and Wingfield 2013; Chang et al. 2019). Additionally, marker genes used for phylogenetic identification frequently vary between species complexes (Linnakoski et al. 2016a; Yin et al. 2019). Isolates of ophiostomatoid fungi recovered from *Cr.piceae* in Japan were exclusively identified using morphological characters (Ohtaka et al. 2002a; Ohtaka et al. 2002b; Yamaoka et al. 2004). On the other hand, those from Poland were either based on ITS sequences (Jankowiak and Kolarik 2010) or ITS, LSU, BT and EF sequences (Jankowiak et al. 2017a). Therefore, the chances of misidentification are high, which can also influence the reported diversity of ophiostomatoid fungi associated with *Cr.piceae* from these regions.

In the last decade, more than a hundred ophiostomatoid fungi have been reported from China. Among these, almost half were previously undescribed species (Yin et al. 2016; Chang et al. 2017; Wang et al. 2018; Chang et al. 2019; Chang et al. 2020; Wang et al. 2020). Owing to climate change, the economic damage caused by these bark beetles and nematodes has exponentially increased in China (Li 2013; Tang et al. 2021), initiating studies focusing on the biology and control of these beetles (Sun et al. 2013). These studies simultaneously cataloged the diversity of symbiotic fungi associated with these beetles, influencing fungal species discovery (Sun et al. 2013; Zhao and Sun 2017).

In this study, we recovered seven species of ophiostomatoid fungi, including five previously undescribed species from the Shandong province of China. The previous study from Shandong province reported two new ophiostomatoid fungi associated with *B.xylophilus* and *M.alternatus* collected from two pine species (Wang et al. 2018). Thus far, more than 10 bark beetle species have been reported from this province (Bai 1985; Zhu et al. 1991). Prior to this study, no attempts were made to isolate ophiostomatoid fungi from the Shandong province of China. Therefore, in the future, follow-up surveys and isolations from other bark beetle species from the province will likely allow the discovery of several novel ophiostomatoid fungi.

## Supplementary Material

XML Treatment for Ceratocystiopsis yantaiensis

XML Treatment for Ceratocystiopsis weihaiensis

XML Treatment for Graphilbum translucens

XML Treatment for Graphilbum niveum

XML Treatment for Sporothrix villosa

## Supplementary materials

Supplementary material 1 Ophiostomatoid fungi associated with *Cryphaluspiaceae* in Shandong province in eastern China Data type molecular data Explanation note Maximum likelihood phylogeny of *Graphium* using complete ITS and partial EF gene regions. The isolates recovered in this study are highlighted in color and in bold font. ML and MP bootstrap support values ≥ 75 are indicated at the nodes as ML/MP. Bold branches indicate posterior probabilities values ≥ 0.9. T indicates ex-type cultures. https://binary.pensoft.net/file/598901This dataset is made available under the Open Database License (http://opendatacommons.org/licenses/odbl/1.0/). The Open Database License (ODbL) is a license agreement intended to allow users to freely share, modify, and use this Dataset while maintaining this same freedom for others, provided that the original source and author(s) are credited.Runlei Chang, Xiuyu Zhang, Hongli Si, Guoyan Zhao, Xiaowen Yuan, Tengteng Liu, Tanay Bose, Meixue Dai

Supplementary material 2 Figure S2 Data type phylogenetic data Explanation note Maximum likelihood phylogeny of *Ophiostomaips* species complex using complete ITS and partial BT gene regions. The isolates recovered in this study are highlighted in color and in bold font. ML and MP bootstrap support values ≥ 75 are indicated at the nodes as ML/MP. Bold branches indicate posterior probabilities values ≥ 0.9. T indicates ex-type cultures. https://binary.pensoft.net/file/598902This dataset is made available under the Open Database License (http://opendatacommons.org/licenses/odbl/1.0/). The Open Database License (ODbL) is a license agreement intended to allow users to freely share, modify, and use this Dataset while maintaining this same freedom for others, provided that the original source and author(s) are credited.Runlei Chang, Xiuyu Zhang, Hongli Si, Guoyan Zhao, Xiaowen Yuan, Tengteng Liu, Tanay Bose, Meixue Dai

Supplementary material 3 Table S1 Data type phylogenetic data Explanation note List of ophiostomatioid fungi used for phylogenetic analyses. T = ex-type culture. https://binary.pensoft.net/file/598903This dataset is made available under the Open Database License (http://opendatacommons.org/licenses/odbl/1.0/). The Open Database License (ODbL) is a license agreement intended to allow users to freely share, modify, and use this Dataset while maintaining this same freedom for others, provided that the original source and author(s) are credited.Runlei Chang, Xiuyu Zhang, Hongli Si, Guoyan Zhao, Xiaowen Yuan, Tengteng Liu, Tanay Bose, Meixue Dai
