# Supplementary figures and images for: Ophiostomatoid species associated with pine trees (Pinus spp.) infested by Cryphaluspiceae from eastern China, including five new species
Source: MycoKeys. 2021 Oct 13;83:181–208. doi: 10.3897/mycokeys.83.70925 (PMC8528803; doi:10.3897/mycokeys.83.70925)

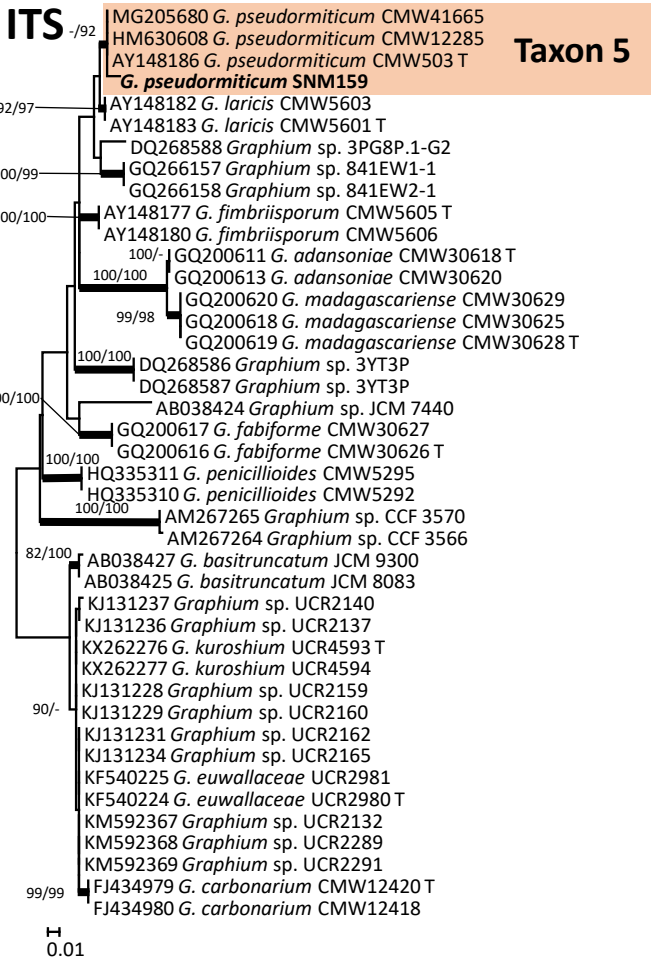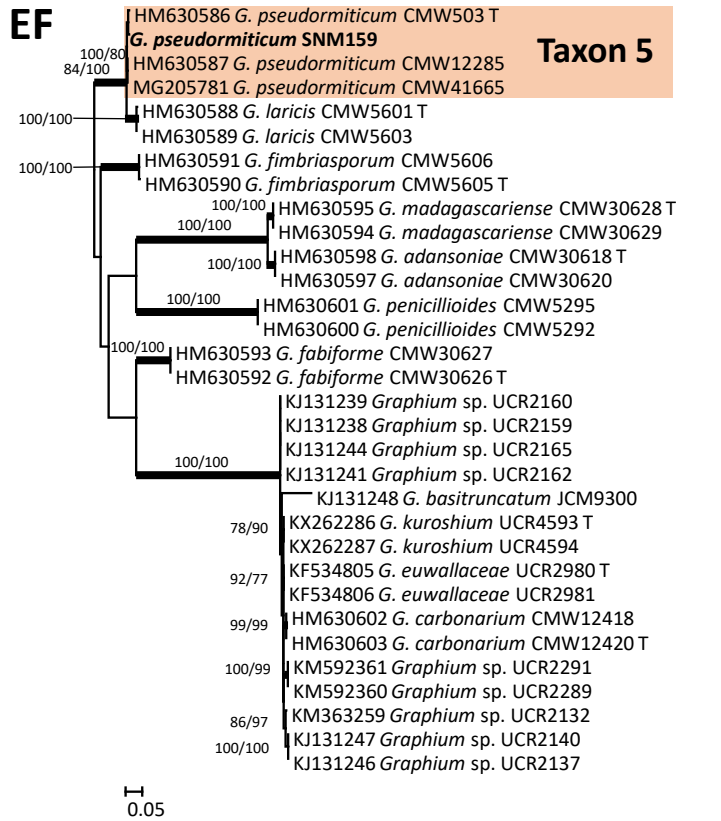

Supplement: Supplementary material 1 — Ophiostomatoid fungi associated with Cryphaluspiaceae in Shandong province in eastern China [file mycokeys-83-181-s001.pdf]

ITS

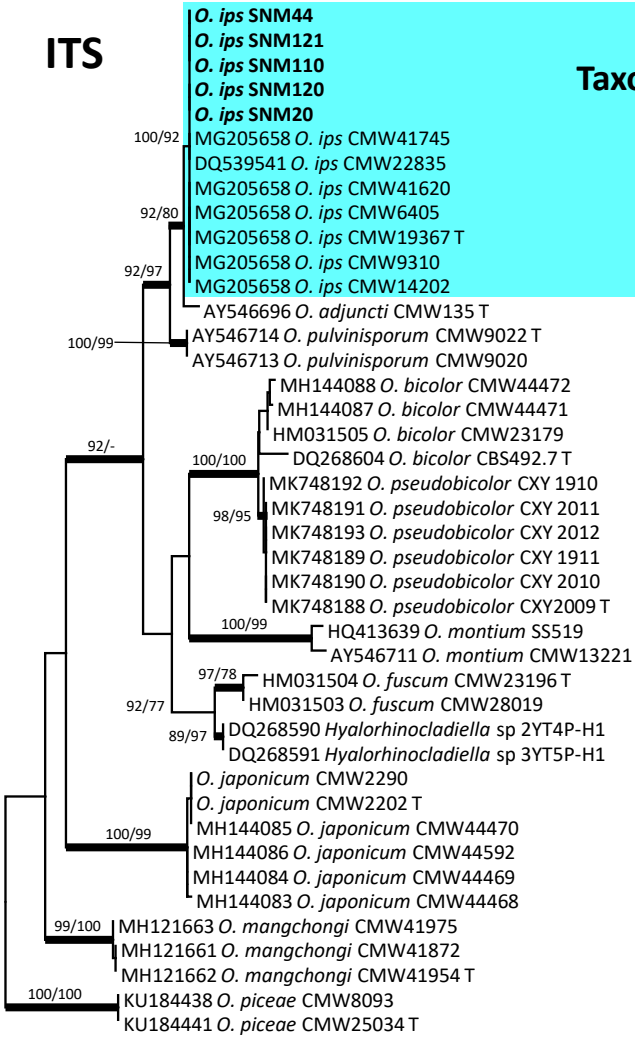

0.01

BT

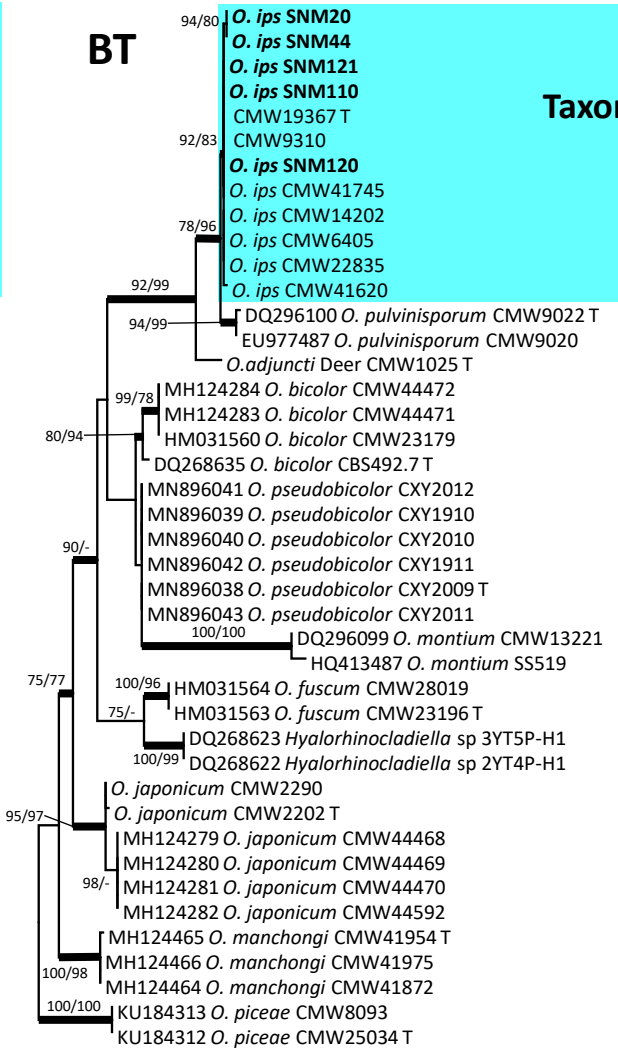

0.01

Supplement: Supplementary material 2 — Figure S2 [file mycokeys-83-181-s002.pdf]
