# Supplementary material for: Ophiostomatoid species associated with pine trees (Pinus spp.) infested by Cryphaluspiceae from eastern China, including five new species
Source: MycoKeys. 2021 Oct 13;83:181–208. doi: 10.3897/mycokeys.83.70925 (PMC8528803; doi:10.3897/mycokeys.83.70925)
Supplement: Supplementary material 3 — Table S1 [file mycokeys-83-181-s003.docx]

Table S1: List of ophiostomatioid fungi used for phylogenetic analyses. T= ex-type culture.

| Species | Isolate | | | GenBank accession numbers of gene regions | | |
| --- | --- | --- | --- | --- | --- | --- |
|  |  | ITS | BT | EF | CAL | LSU |
| *Ceratocystiopsis brevicomis* | UM1452 | EU913722 | EU913761 | - | - | EU913683 |
|  | CQ05 | - | KJ620531 | - | - | - |
|  | GO05 | - | KJ620530 | - | - | - |
|  | CBS137839 | - | - |  |  | MW028162 |
| *C. collifera* | CBS 126.89 | MH862160 | EU913760 | - | - | EU913681 |
| *C. concentrica* | WIN(M)71-07 | - | - |  |  | AF135571 |
| *C. lunata* | CMW55897^T^ | MW028169 | MW066754 | - | - | - |
|  | CMW55898 | MW028170 | MW066755 | - | - | - |
| *C. manitobense* | 3YT3P-Om | DQ268609 | DQ268641 | - | - | - |
|  | 3PG2P-Om | DQ268610 | DQ268640 | - | - | - |
| *C. manitobensis* | UM214 | EU913715 | DQ268639 | - | - | EU913675 |
|  | UM237 | EU913714 | EU913753 | - | - | EU913674 |
| *C. minima* | UM235 | EU913702 | EU913741 | - | - | EU913661 |
|  | UM85 | EU913701 | EU913740 | - | - | EU913660 |
| *C. minuta* | CBS 116796 | EU913695 | EU913734 | - | - | EU913654 |
|  | UM1535^T^ | EU913700 | EU913739 | - | - | EU913656 |
| *C. minuta-bicolor* | UM844 | EU913706 | EU913745 | - | - | EU913665 |
|  | UM480 | EU913705 | EU913744 | - | - | - |
|  | CBS 635.66 | MH858901 | EU977482 | - | - | MH870571 |
| *C. pallidobrunnea* | WIN(M)51 | MN901004 | MN901013 | - | - | - |
|  | UM51 | - | - |  |  | EU913682 |
| *C. ranaculosa* | CBS 216.88 | MH862126 | EU913752 | - | - | EU913673 |
|  | 949-P320 | MT875423 | - | - | - | - |
|  | C1245 | - | AY548746 | - | - | - |
|  | CMW13940 | - | - |  |  | DQ294357 |
| *C. rollhanseniana* | UM110 | EU913719 | EU913758 | - | - | EU913679 |
|  | UM113 | EU913718 | EU913757 | - | - | EU913678 |
| *C. synnemata* | KFL17718DA | MN900989 | MN901010 | - | - | - |
|  | KFL16918DA | MN900988 | MN901009 | - | - | - |
| *C. weihaiensis* sp. nov. | SNM634 | MW989412 | MZ019524 | MZ853081 | - | MZ819925 |
|  | SNM649^T^=CGMCC3.20246 | MW989413 | MZ019525 | MZ853082 | - | MZ819926 |
| *C. yantaiensis* sp. nov. | SNM582 | MW989410 | MZ019522 | MZ853079 | - | MZ819923 |
|  | SNM650^T^=CGMCC3.20247 | MW989411 | MZ019523 | MZ853080 | - | MZ819924 |
| *Ceratocystiopsis* sp. | WY13TX1-3 | EU913707 | EU913746 | - | - | EU913667 |
|  | WY21TX1-2 | EU913708 | EU913747 | - | - | - |
|  | WY21TX2-2 | EU913709 | EU913748 | - | - | - |
|  | YCC330 | EU913710 | EU913749 | - | - | EU913670 |
|  | YCC329 | EU913711 | EU913750 | - | - | EU913671 |
| *Ophiostoma bacillisporum* | CBS 771.71^T^=MUCL 44874 | MH860346 | MH055732 | - | - | MH872100 |
| *Graphilbum anningense* | CXY1944 | MH555902 | MH683597 | - | - | - |
|  | CXY1939^T^ | MH555903 | MH683595 | - | - | - |
|  | CXY1940 | MH555901 | MH683596 | - | - | - |
| *Gr. acuminatum* | CBS 145825 | MN548900 | MN548936 | MN548950 | MN548989 |  |
|  | CBS 145811 | MN548899 | MN548935 | MN548949 | - | - |
|  | 110bPRJ | KU319038 | - | KU319184 | - |  |
|  | CBS 145827 | MN548901 | MN548937 | MN548951 | MN548990 | - |
|  | CBS 145810 | MN548898 | MN548934 | MN548948 | - | - |
|  | CBS 145828^T^ | MN548902 | MN548938 | MN548952 | MN548991 | - |
|  | CBS 145809 | MN548897 | MN548933 | MN548947 | - | - |
|  | CBS 145826 | - | MN548939 | - | - | - |
| *Gr. carpaticum* | CBS 145837 | MN548903 | MN548940 | MN548957 | MN548997 | - |
|  | 143aMFJD^T^=CBS 145835 | KY568116 | KY568226 | MN548954 | MN548996 | - |
|  | 132aMFJD | KY568118 | KY568228 | - | - | - |
|  | CBS 145834 | - | - | MN548955 | MN548995 | - |
| *Gr. crescericum* | CBS 145824 | MN548929 | - | MN548982 | MN549020 | - |
|  | CBS 145822 | MN548931 | - | MN548984 | MN549022 | - |
|  | CBS 145821 | MN548928 | - | MN548981 | MN549019 | - |
|  | CBS 145823 | MN548932 | - | MN548985 | MN549023 | - |
|  | CBS 145820 | MN548927 | - | MN548980 | MN549018 | - |
|  | N2015-0763/2/1 | MN548930 | - | MN548983 | MN549021 |  |
|  | CBS 130864 | MN548925 | - | MN548978 | MN549016 | - |
|  | CBS 130866 | MN548926 | - | MN548979 | MN549017 | - |
|  | 17114aFJD | - | - | KY568504 | - | - |
| *Gr. curvidentis* | 17814aFJD | KY568112 | - | KY568503 | MN549001 |  |
|  | 55KFJD^T^=CBS145832 | KY568111 | - | KY568502 | MN549000 | - |
| *Gr. fragrans* | CBS 27954 ^T^ | AF198248 | - | - | - | - |
|  | 44aMFJD | KY568103 | KY568217 | KY568494 | - | - |
|  | 9214FJD | KY568106 | KY568220 | KY568497 | - | - |
|  | CMW43200 | - | - | - | MN549024 | - |
|  | CMW44159 | - | - | - | MN549025 | - |
| *Gr. furuicola* | CBS 145812 | MN548908 | - | MN548962 | MN549006 | - |
|  | CBS 145836 | MN548906 | - | MN548960 | MN549004 | - |
|  | CBS 145813^T^ | MN548907 | - | MN548961 | MN549005 | - |
| *Gr. gorcense* | CMW34152 | MN548918 | MN548944 | MN548971 | - | - |
|  | CMW34151 | MN548917 | MN548943 | MN548970 | - | - |
|  | CMW34153^T^ | MN548919 | MN548945 | MN548972 | - | - |
| *Gr. interstitiale* | CBS 145818 | MN548911 | - | MN548965 | MN549009 | - |
|  | CBS 145817 | MN548910 | - | MN548964 | MN549008 | - |
|  | CBS 145816^T^ | MN548909 | - | MN548963 | MN549007 | - |
| *Gr. ipis-grandicollis* | VPRI43761 | MW046070 | MW066358 | MW066404 | MW075119 |  |
|  | VPRI43762^T^ | MW046071 | MW066359 | MW066405 | MW075120 |  |
| *Gr. kesiyae* | CMW41729^T^ | MG205669 | MG205713 | OK165560 | - | - |
|  | CMW41657 | MG205668 | MG205714 | OK165561 | - | - |
| *Gr. microcarpum* | YCC459 | AB506676 | - | - | - | - |
| *Gr. nigrum* | CBS 163.61 | MH858010 | - | - | - | - |
| *Gr. niveum* sp. nov. | SNM100 | MW989417 | MZ019529 | MZ019547 | MZ418998 | - |
|  | SNM145^T^=CGMCC3.20268 | MW989418 | MZ019530 | MZ019548 | MZ418997 | - |
| *Gr. puerense* | CMW41667 | MG205670 | MG205716 | OK165562 | - | - |
|  | CMW41942^T^ | MG205671 | MG205719 | OK165563 | - | - |
|  | CMW41619 | MG205670 | MG205715 | OK165564 | - | - |
|  | CMW41998 | MG205670 | MG205715 | OK165565 | - | - |
|  | CMW41971 | MG205671 | MG205720 | OK165566 | - | - |
|  | CMW41996 | MG205671 | MG205721 | OK165567 | - | - |
|  | CMW41671 | MG205670 | MG205717 | OK165568 | - | - |
|  | CMW41673 | MG205670 | MG205718 | OK165569 | - | - |
| *Gr. rectangulosporium* | TFM FPH 7756^T^ | AB242825 | - | - | - | - |
| *Gr. roseum* | T46 | KY050751 | - | - | - | - |
|  | T79 | KY050752 | - | - | - | - |
|  | T72 | KY050753 | - | - | - | - |
| *Gr. sexdentatum* | CBS 145814^T^ | MN548915 | - | MN548968 | MN549013 | - |
|  | N2015-1067/1/4 | MN548914 | - | - | MN549012 | - |
|  | N2015-1087/2/1 | MN548916 | - | MN548969 | - | - |
|  | CBS 145815 | - | - | MN548967 | MN549011 | - |
| *Gr. sparsum* | CBS 405.77^T^ | MN548924 | MN548946 | MN548977 | MN549015 | - |
|  | N2015-0869/2/2 | MN548922 | - | MN548975 | - | - |
|  | CMW54772 | MN548923 | - | MN548976 | - | - |
| *Gr. translucens* sp. nov. | SNM101 | MW989414 | MZ019526 | MZ019544 | MZ781969 | - |
|  | SNM104 | MW989415 | MZ019527 | MZ019545 | MZ781970 | - |
|  | SNM144^T^=CGMCC3.20263 | MW989416 | MZ019528 | MZ019546 | MZ781971 | - |
| *Gr. tsugae* | UAMH 11701 | KJ661745 | - | - | - | - |
| *Graphilbum* sp. 3 | 17114aFJD | KY568119 | - | - | - | - |
| *Sporothrix dentifunda* | CMW13016 | AY495434 | AY495445 | - | - | - |
|  | KFL408DB16bRJ | MH740954 | MH741090 | MH741182 | MH741220 | - |
|  | CBS 115790^T^ | - | - | - | KX590787 | - |
| *S. aurorae* | CMW19362^T^=CBS 118837 | DQ396796 | DQ396800 | - | KX590783 | - |
|  | CMW19363 | DQ396797 | DQ396801 | - | - | - |
| *S. cantabriensis* | CMW39768 | KF951556 | KF951546 | - | KF951542 | - |
|  | CMW39766^T^ | KF951554 | KF951544 | - | KF951540 | - |
|  | CMW39767 | KF951554 | KF951545 | - | KF951541 | - |
| *S. cf. abietina* | CMW109 | AY280487 | AY280469 | - | - | - |
|  | CMW110 | AY280488 | AY280470 | - | - | - |
|  | CMW26269 | EU785445 | EU785433 | - | JQ511963 | - |
|  | CMW26262 | EU785446 | EU785434 | - | JQ511964 | - |
|  | CBS12589^T^ = CMW22310 | AF484453 | HM067820 | - | JQ511966 | - |
|  | C201 | - | AY789149 | - | - | - |
|  | CMW1468 | - | AY280468 | - | - | - |
|  | DLS1340 | - | DQ865287 | - | - | - |
|  | NZFS 1556 | - | AY789148 | - | - | - |
|  | KUC 2671 | - | AY789147 | - | - | - |
| *S. cracoviensis* | CBS147941 | MW768963 | MH283367 | - | MH283528 | - |
|  | CBS147942^T^ | MW768964 | MH283368 | - | MH283529 | - |
| *S. curviconia* | CMW17163^T^ | KX590836 | - | - | JQ511968 | - |
|  | CBS 541.84 | - | KX590777 | - | - | - |
| *S. eucastanea* | CBS 424.77^T^ | KX590814 | KX590753 | - | KX590781 | - |
|  | KFL1141N16DBRJ | MH740955 | MH741091 | - | MH741221 | - |
| *S. euskadiensis* | CMW27898 | DQ674370 | GU566608 | - | JQ438831 | - |
|  | CMW27318^T^ | DQ674369 | EF396344 | - | JQ438830 | - |
|  | CMW27899 | DQ674371 | GU566609 | - | JQ438832 | - |
| *S. fraxini* | CBS147936^T^ | MH283150 | MH283370 | - | MH283530 | - |
|  | CBS147938 | MW768968 | MH283371 | - | MH283531 | - |
| *S. fusiformis* | CMW8281 | AY280482 | AY280462 | - | - | - |
|  | CMW9968^T^ | AY280481 | AY280461 | - | JQ511967 | - |
|  | CMW8285 | AY280483 | AY280463 | - | - | - |
|  | CMW10565 | AY280484 | AY280465 | - | - | - |
|  | CMW7131 | - | - | - | JQ511971 | - |
|  | KFL43916RJSR | - | - | - | MH283518 | - |
| *S. gossypina* | ATCC 18999^T^ | KX590819 | KX590761 | - | KX590789 | - |
| *S. lunata* | CMW10563^T^ | AY280485 | AY280466 | - | JQ511970 | - |
|  | CMW10564 | AY280486 | AY280467 | - | - | - |
| *S. nebularis* | CMW27319^T^ | MG205654 | OK165570 | - | OK165572 | - |
|  | CMW27900 | MG205655 | OK165571 | - | OK165573 | - |
| *S. prolifera* | CBS 251.88^T^ | KX590829 | KX590770 | - | KX590797 | - |
|  | KFL218N16  TARAO | MH740956 | MH741093 | - | MH741222 | - |
| *S. rossii* | CBS 116.78^T^ | KX590815 | KX590754 | - | - | - |
| *S. uta* | CMW40318^T^ | KU595576 | KU639618 | - | KU639607 | - |
|  | CMW40316 | KU595577 | KU639616 | - | KU639605 | - |
|  | CMW40317 | KU595578 | KU639617 | - | KU639606 | - |
| *S. variecibatus* | CMW23051^T^ | DQ821568 | DQ821539 | - | - | - |
|  | CMW23060 | DQ821569 | DQ821573 | - | KP101479 | - |
|  | CBS 121961 | - | - | - | KX590813 | - |
| *S. villosa* sp. nov. | SNM162 | MW989425 | MZ019536 | MZ853075 | MZ019540 | - |
|  | SNM182 | MW989426 | MZ019537 | MZ853076 | MZ019541 | - |
|  | SNM185 | MW989427 | MZ019538 | MZ853077 | MZ019542 | - |
|  | SNM188^T^=CGMCC3.20264 | MW989428 | MZ019539 | MZ853078 | MZ019543 | - |
| *Graphium adansoniae* | CMW30618^T^ | GQ200611 | - | HM630598 | - | - |
|  | CMW30620 | GQ200613 | - | HM630597 | - | - |
| *G. basitruncatum* | JCM 9300 | AB038427 | - | KJ131248 | - | - |
|  | JCM 8083 | AB038425 | - | - | - | - |
| *G. carbonarium* | CMW12420^T^ | FJ434979 | - | HM630603 | - | - |
|  | CMW12418 | FJ434980 | - | HM630602 | - | - |
| *G. euwallaceae* | UCR2981 | KF540225 | - | KF534806 | - | - |
|  | UCR2980^2^ | KF540224 | - | KF534805 | - | - |
| *G. fabiforme* | CMW30627 | GQ200617 | - | HM630593 | - | - |
|  | CMW30626^T^ | GQ200616 | - | HM630592 | - | - |
| *G. fimbriisporum* | CMW5605^T^ | AY148177 | - | HM630590 | - | - |
|  | CMW5606 | AY148180 | - | HM630591 | - | - |
| *G. kuroshium* | UCR4593^T^ | KX262276 | - | KX262286 | - | - |
|  | UCR4594 | KX262277 | - | KX262287 | - | - |
| *G. laricis* | CMW5603 | AY148182 | - | HM630589 | - | - |
|  | CMW5601^T^ | AY148183 | - | HM630588 | - | - |
| *G. madagascariense* | CMW30629 | GQ200620 | - | HM630594 | - | - |
|  | CMW30625 | GQ200618 | - | - | - | - |
|  | CMW30628^T^ | GQ200619 | - | HM630595 | - | - |
| *G. penicillioides* | CMW5295 | HQ335311 | - | HM630601 | - | - |
|  | CMW5292 | HQ335310 | - | HM630600 | - | - |
| *G. pseudormiticum* | CMW41665 | MG205680 | - | MG205781 | - | - |
|  | CMW12285 | HM630608 | - | HM630587 | - | - |
|  | CMW503^T^ | AY148186 | - | HM630586 | - | - |
|  | SNM159 | MW989419 | - | MZ019549 | - | - |
| *Graphium* sp. | 3PG8P.1-G2 | DQ268588 | - | - | - | - |
|  | 841EW1-1 | GQ266157 | - | - | - | - |
|  | 841EW2-1 | GQ266158 | - | - | - | - |
|  | 3YT3P.1-G1 | DQ268586 | - | - | - | - |
|  | 3YT3P.2-G1 | DQ268587 | - | - | - | - |
|  | JCM 7440 | AB038424 | - | - | - | - |
|  | CCF 3570 | AM267265 | - | - | - | - |
|  | CCF 3566 | AM267264 | - | - | - | - |
|  | UCR2140 | KJ131237 | - | KJ131247 | - | - |
|  | UCR2137 | KJ131236 | - | KJ131246 | - | - |
|  | UCR2159 | KJ131228 | - | KJ131238 | - | - |
|  | UCR2160 | KJ131229 | - | KJ131239 | - | - |
|  | UCR2162 | KJ131231 | - | KJ131241 | - | - |
|  | UCR2165 | KJ131234 | - | KJ131244 | - | - |
|  | UCR2132 | KM592367 | - | KM592359 | - | - |
|  | UCR2289 | KM592368 | - | KM592360 | - | - |
|  | UCR2291 | KM592369 | - | KM592361 | - | - |
| *O. adjuncti* | CMW135^T^ | AY546696 | - | - | - | - |
| *O. bicolor* | CMW44472 | MH144088 | MH124284 | - | - | - |
|  | CMW44471 | MH144087 | MH124283 | - | - | - |
|  | CMW44479 | HM031505 | HM031560 | - | - | - |
|  | CBS492.7^T^ | DQ268604 | DQ268635 | - | - | - |
| *O. fuscum* | CMW23196^T^ | HM031504 | HM031563 | - | - | - |
|  | CMW28019 | HM031503 | HM031564 | - | - | - |
| *O. ips* | SNM20 | MW989420 | MZ019531 | - | - | - |
|  | SNM44 | MW989421 | MZ019532 | - | - | - |
|  | SNM110 | MW989422 | MZ019533 | - | - | - |
|  | SNM120 | MW989423 | MZ019534 | - | - | - |
|  | SNM121 | MW989424 | MZ019535 | - | - | - |
|  | CMW41745 | MG205658 | MG205688 | - | - | - |
|  | CMW41695 | MG205658 | MG205688 | - | - | - |
|  | CMW41620 | MG205658 | MG205687 | - | - | - |
|  | CMW41697 | MG205658 | MG205688 | - | - | - |
|  | CMW41709 | MG205658 | MG205688 | - | - | - |
|  | CMW41916 | MG205658 | MG205688 | - | - | - |
|  | CMW41993 | MG205658 | MG205688 | - | - | - |
| *O. japonicum* | CMW44470 | MH144085 | MH124281 | - | - | - |
|  | CMW44592 | MH144086 | MH124282 | - | - | - |
|  | CMW44469 | MH144084 | MH124280 | - | - | - |
|  | CMW44468 | MH144083 | MH124279 | - | - | - |
| *O. mangchongi* | CMW41975 | MH121663 | MH124466 | - | - | - |
|  | CMW41872 | MH121661 | MH124464 | - | - | - |
|  | CMW41954^T^ | MH121662 | MH124465 | - | - | - |
| *O. montium* | SS519 | HQ413639 | HQ413487 | - | - | - |
|  | CMW13221 | AY546711 | DQ296099 | - | - | - |
| *O. piceae* | CMW8093 | KU184438 | KU184313 | - | - | - |
|  | CMW25034^T^ | KU184441 | KU184312 | - | - | - |
| *O. pseudobicolor* | CXY 1910 | MK748192 | MN896039 | - | - | - |
|  | CXY 2011 | MK748191 | MN896043 | - | - | - |
|  | CXY 1012 | MK748193 | MN896041 | - | - | - |
|  | CXY 1911 | MK748189 | MN896042 | - | - | - |
|  | CXY 2010 | MK748190 | MN896040 | - | - | - |
|  | CXY 2009^T^ | MK748188 | MN896038 | - | - | - |
| *O. pulvinisporum* | CMW9022^T^ | AY546714 | DQ296100 | - | - | - |
|  | CMW9020 | AY546713 | EU977487 | - | - | - |
| *Hyalorhinocladiella* sp. | 2YT4P-H1 | DQ268590 | DQ268622 | - | - | - |
|  | 3YT5P-H1 | DQ268591 | DQ268623 | - | - | - |
